# Supplementary material for: Breast hypoplasia markers among women who report insufficient milk production: A retrospective online survey
Source: PLoS One. 2024 Feb 29;19(2):e0299642. doi: 10.1371/journal.pone.0299642 (PMC10903845; doi:10.1371/journal.pone.0299642)
Supplement: S1 Checklist — (DOCX) [file pone.0299642.s001.docx]

STROBE Statement—checklist of items that should be included in reports of observational studies

|  | Item No. | Recommendation | Page  No. | Relevant text from manuscript |
| --- | --- | --- | --- | --- |
| **Title and abstract** | 1 | (*a*) Indicate the study’s design with a commonly used term in the title or the abstract | 1 | retrospective online survey |
|  |  | (*b*) Provide in the abstract an informative and balanced summary of what was done and what was found | 2-3 | **Objectives:** To estimate the proportions of anatomical breast characteristics suggestive of breast hypoplasia among breastfeeding women self-reporting low milk supply. We also explored breast hypoplasia risk factors.  **Design:** Online survey conducted between October 2021 and January 2022.  **Setting:** Low milk supply Facebook groups.  **Participants:** 487 women reporting low milk supply with their index child born ≥ 37 weeks gestation within 5 years of participation in this study, and residing in the USA, Australia or the UK. We present data on the primary outcome (‘breast type’) for 399 women. Women were excluded if the dyad was separated for more than 24 hours during the hospital stay, or if the mother reported removing milk for less than 6 times per day from each breast on most days before being aware of having insufficient milk production.  **Primary and secondary outcome measures:** The proportions of proposed breast hypoplasia markers including atypical breast type, widely spaced breasts, breast asymmetry, stretch marks on the breast and lack of pregnancy breast growth. We also estimated the odds of having breast hypoplasia markers in at-risk groups compared to reference groups, adjusting for covariates.  **Results:** Approximately 68% reported at least one atypical breast (270/399; 95% CI: 62.9%, 72.1%). Around 47% reported widely spaced breasts (212/449; 95% CI: 42.7%, 52.7%), 72% a lack of pregnancy breast growth (322/449; 95% CI: 68.3%, 77.4%), and 76% stretch marks on the breast (191/250; 95% CI: 70.7%, 81.3%). Multiple logistic regression analyses identified high BMI as a risk factor for widely spaced breasts, stretch marks on the breast and lack of pregnancy breast growth.  **Conclusions:** Participants in low milk supply Facebook groups reported high rates of breast hypoplasia markers. High BMI is a risk factor for breast hypoplasia markers. These findings need to be confirmed in well-conducted large cohort studies to determine the strongest combination of hypoplasia markers in predicting low supply. |
| Introduction | | | |  |
| Background/rationale | 2 | Explain the scientific background and rationale for the investigation being reported | 4-6 | Although most new mothers commence breastfeeding, many cite low milk supply as the reason for stopping breastfeeding prematurely [1-3]. A mother experiences primary insufficient milk supply when her body cannot produce enough milk to enable exclusive breastfeeding despite regular milk removal [4]. One possible reason for primary insufficient milk production is breast hypoplasia [5]. When breast hypoplasia is present, there is a lack of sufficient glandular tissue [6].  Breast hypoplasia can be congenital or acquired [7]. Congenital breast hypoplasia is associated with uncommon syndromes (e.g. Poland or Jeune) or chest wall deformities (e.g. pectus excavatum) [7]. Acquired breast hypoplasia can be associated with a history of breast radiation or breast haemangioma [7]. Other acquired cases of breast hypoplasia have no identifiable cause, although pubertal and/or gestational glandular tissue development may be hampered by various endocrine alterations [8-17].  There is a lack of research investigating possible links between breast anatomical variations and lactation outcomes. Ventura et al found that among women with “more dense areolae”, shorter and wider nipples were associated with a greater chance of experiencing low milk supply and slow infant weight gain [18]. A study by Vazirinejad and colleagues determined that infants of mothers with breast variations (any form of “large nipple”, “flat nipple”, “inverted nipple” and “abnormally large breast”) had significantly lower weight gain than infants of mothers without these variations [19]. Other researchers found that no or slight pregnancy breast growth, and no or slight postpartum breast engorgement with secretory activation (lactogenesis II or “milk coming in”), were associated with inadequate infant weight gain and shorter breastfeeding duration [8,20].  Prior to our research, the largest study to investigate a possible relationship between anatomical breast characteristics suggestive of breast hypoplasia and milk production was a case series of 34 women conducted by Huggins et al [21]. These researchers adapted a tool from a retrospective analysis of 40 patients undergoing operative breast corrections [22] to categorise women's breasts into one of four types (Fig in S1 Fig) [21]. In their sample, the women’s ‘breast type’ appeared to be related to the adequacy of their milk production, as women with type 2, 3 or 4 breasts produced insufficient milk compared to women with type 1 (typical appearance) breasts [21].  Huggins and colleagues also identified other anatomical breast characteristics they suspected were associated with primary insufficient milk production due to breast hypoplasia [21]:   - Noticeable breast asymmetry (i.e., a marked difference in size, or size and shape, of the breasts); - A wide intermammary width (≥ 3.8 cm or 1.5 inches), because of underdevelopment of the inner aspect of the breast; - Stretch marks on one or both breasts (Huggins et al observed their presence when evaluating breast hypoplasia); - Little or no pregnancy breast growth, which may suggest atypical mammogenesis; - A lack of breast fullness in the first week postpartum which may indicate a deficiency in secretory differentiation in pregnancy and/or secretory activation (lactogenesis II) after giving birth.   No prior research has explored the prevalence of different breast types or proposed markers of breast hypoplasia among women with low milk production. Also, the feasibility of asking women to self-report their own anatomical breast characteristics has not previously been examined. Because of the importance of breastfeeding for maternal and infant health [23], it is important to elucidate the role maternal breast anatomy plays in low milk production. Therefore, using an online survey of women self-identifying as having low milk supply, we aimed to estimate the proportion of women with various anatomical breast characteristics related to breast hypoplasia, assess the feasibility of maternal self-report of these characteristics and to explore breast hypoplasia risk factors. |
| Objectives | 3 | State specific objectives, including any prespecified hypotheses | 6-7 | **Objectives**  **Primary objective**  The primary objective was to estimate the proportion of women with self-reported low milk production describing at least one breast as type 2, 3 or 4 as per Huggins et al (Fig in S1 Fig) [21]. In this paper we refer to a type 2, 3 or 4 breast as an ‘atypical’ breast type and a type 1 breast as a ‘typical’ breast type.  **Secondary objectives**  Secondary aims included determining the feasibility of asking women to self-report their breast anatomy characteristics and assessing the proportions of proposed markers of breast hypoplasia including a wide space between the breasts (referred to as ‘intramammary space’ by Huggins et al.) (≥ 1.5 inches or 3.8 cm) [21]; lack of breast growth in their index pregnancy (no noticeable change in or an increase of < 1 bra cup size to either breast during index pregnancy); presence of stretch marks on one or both breasts [21] prior to birth of first child; and breast asymmetry (≥ 2 cup size difference between their breasts).  In addition, we aimed to determine the proportion of women with delayed secretory activation (breasts becoming noticeably fuller > 72 hours postpartum [24,25] or breasts never became noticeably fuller with participants’ index child).  We also planned to explore associations between endocrine conditions (polycystic ovary syndrome, diabetes, hypothyroidism as well as high BMI) and at least one breast being atypical; whether a link exists between the endocrine conditions listed above and proposed markers of breast hypoplasia (listed above); if proposed markers of breast hypoplasia are associated with breast type; and whether a relationship exists between having at least one atypical breast and delayed secretory activation. |
| Methods | | | |  |
| Study design | 4 | Present key elements of study design early in the paper | 7 | **Design**  We conducted an open voluntary retrospective online survey of women belonging to low milk production Facebook support groups [26,27]. This design enabled us to recruit participants and conduct research at a time when face-to-face research was limited due to the COVID-19 pandemic. It also enabled timely recruitment of participants in our target population: women with low milk supply. This study received approval by the La Trobe University Human Ethics Committee (approved 21 September 2021; approval number HEC21306). |
| Setting | 5 | Describe the setting, locations, and relevant dates, including periods of recruitment, exposure, follow-up, and data collection | 7-10 | **Sample and eligibility criteria**  A convenience sample of women who self-reported low milk supply completed the survey. No incentives were offered for participation. Women were eligible for participation if they typically resided in Australia, the United States or the United Kingdom; were 18 years or older; could read and write in English; and reported low milk supply with their first live birth of a term singleton (≥ 37.0 weeks gestation) born within 5 years of participation in the study. In order to reduce secondary causes of low milk production, exclusion criteria included mother or index infant requiring special care during the birth hospitalisation resulting in dyad separation for more than 24 hours; or if the mother reported not removing milk at least 6 times per day from each breast on most days prior to being aware of having insufficient milk production with the index infant.  **Patient and public involvement**  While community members were not directly involved in designing or conducting this project, the investigators used their experience in caring for women with low milk supply (RLK, LHA, JI) and working with patient support groups (LNR) to inform the research questions and analyses.  **Survey**  Previously, we devised a survey and diagram depicting breast types to conduct a reliability study and confirmed that researchers could reliably measure women’s intermammary width [28]. The survey for this current study was designed by adapting items from the questionnaire used in the reliability study [28], with the addition of several new questions investigating anatomical breast characteristics. The breast type classification (primary outcome) item was based on the breast type diagram devised for the reliability study (Fig 1) [28]. All survey questions were piloted with the research team (n=5), research colleagues (n=10) and a small group of similar women (n=7) in an iterative manner.  The survey was launched and shared in low milk supply Facebook groups as well as via the first author’s personal and business (lactation consultant) Facebook pages. Keywords used to search Facebook for low milk supply support groups included “breast hypoplasia”, “insufficient glandular tissue”, “supply line” or “low milk supply”. The five low milk supply Facebook support groups where the survey was shared were: ‘IGT And Low Milk Supply Support Group’, ‘Supply Line Breastfeeders Support Group of Australia’, ‘IGT Off Topic Group’, ‘Low Milk Supply - A Mother's Love’ and ‘Low Milk Supply/Domperidone’ (Table in S1 Table). The first author joined each group and contacted the group administrator(s) to provide information about the study and request permission to recruit participants using the Facebook site.  The survey consisted of 78 items organised into a structured online questionnaire with skip logic, and was administered through REDCap, a secure web-based application for data collection and management (File in S1 File) [29,30]. All questions related to participants’ first child. Where relevant, questions had “unsure” and “prefer not to say” as options, and participants were able to skip questions. Depending on skip logic, the survey was up to 21 pages with up to 11 questions per page. Participants could go back to a previous page to review or change responses if they wished.  Women were screened using an eligibility survey (File in S2 File) where they were informed about the purpose of the study. Eligible participants could download and read the Participant Information Statement and were asked “Do you agree to complete the survey? Clicking 'Yes' tells us you want to take part in the study.” If a participant provided consent to complete the survey by clicking ‘Yes’, they were lead to the survey. We had access to no information that could identify individual participants during or after data collection.  The survey was open for 16 weeks between October 2021 and January 2022. At the opening of the survey, the first author or a group administrator posted a brief description of the study purpose to each Facebook group and provided a link to the REDCap survey. Snowballing was possible as the post may have been shared with other Facebook groups/pages/members. The first author interacted by thanking group members for their participation to help maintain traffic to the posts. Additional posts, again describing study purpose and linking to survey, were made 1-2 more times in the largest two groups (‘IGT and Low Milk Supply Support Group’ and ‘Supply Line Breastfeeders Support Group of Australia’) over the recruiting period until the target sample size was reached. |
| Participants | 6 | (*a*) *Cohort study*—Give the eligibility criteria, and the sources and methods of selection of participants. Describe methods of follow-up  *Case-control study*—Give the eligibility criteria, and the sources and methods of case ascertainment and control selection. Give the rationale for the choice of cases and controls  *Cross-sectional study*—Give the eligibility criteria, and the sources and methods of selection of participants | 7-8 | **Sample and eligibility criteria**  A convenience sample of women who self-reported low milk supply completed the survey. No incentives were offered for participation. Women were eligible for participation if they typically resided in Australia, the United States or the United Kingdom; were 18 years or older; could read and write in English; and reported low milk supply with their first live birth of a term singleton (≥ 37.0 weeks gestation) born within 5 years of participation in the study. In order to reduce secondary causes of low milk production, exclusion criteria included mother or index infant requiring special care during the birth hospitalisation resulting in dyad separation for more than 24 hours; or if the mother reported not removing milk at least 6 times per day from each breast on most days prior to being aware of having insufficient milk production with the index infant. |
|  |  | (*b*) *Cohort study*—For matched studies, give matching criteria and number of exposed and unexposed  *Case-control study*—For matched studies, give matching criteria and the number of controls per case | N/A |  |
| Variables | 7 | Clearly define all outcomes, exposures, predictors, potential confounders, and effect modifiers. Give diagnostic criteria, if applicable | 10-12 | **Variables**  **Outcome variables**  The primary outcome was the proportion of participants with at least one atypical breast. Participants were asked to indicate what each of their breasts individually looked like just prior to pregnancy with their first child using Figure in S2 Fig. Participants who had breast surgery prior to the birth of their first child were asked to report breast appearance prior to surgery.  A secondary outcome was the feasibility of self-administration of the survey based on the proportion for whom breast type and other individual markers suggestive of breast hypoplasia could be determined. Additional secondary outcomes included the proportion of participants with other individual markers suggestive of breast hypoplasia as well as a delay in secretory activation. We also estimated the odds of having markers of breast hypoplasia in at-risk groups compared to reference groups, adjusting for covariates.  **Exposure variables**  Various endocrine alterations including polycystic ovary syndrome (PCOS), diabetes (type I, II or gestational) and hypothyroidism have been identified as being associated with breast hypoplasia [8-17]. Therefore, participants were asked whether they had any such endocrine conditions medically diagnosed prior to the birth of their first child. Data were collected about the timing of onset of these conditions and medications used to manage a diagnosis of PCOS, gestational diabetes mellitus (GDM) or type II diabetes. Participants were asked to provide estimates of their height and weight just before their index pregnancy in order to calculate their pre-pregnancy BMI. BMI was defined as per the World Health Organization BMI categories [31]: <18.5 kg/m^2^ (underweight), 18.5 to <25.0 kg/m^2^ (normal weight), 25.0 to <30.0 kg/m^2^ (overweight), 30.0 to <35.0 kg/m^2^ (obese class 1), 35.0 to <40.0 kg/m^2^ (obese class 2), and ≥40.0 kg/m^2^ (obese class 3).  **Other covariates**  Demographic characteristics including current age, country of residence, marital status and education were collected. Ethnicity was collected separately for each country where eligible women usually resided in. For participants who typically resided in Australia, questions related to indigeneity and country of birth were asked. Data about intention to breastfeed (by asking how long women planned to breastfeed their baby for) were collected.  Participants were also asked about conditions, obstetric history (method of birth and analgesia used during labour) or surgery which may interfere with mammary glandular tissue development and/or lactation capacity. A final open-ended question was asked about participants’ personal stories of how their low milk supply was discovered or diagnosed (not included in this paper). |
| Data sources/ measurement | 8* | For each variable of interest, give sources of data and details of methods of assessment (measurement). Describe comparability of assessment methods if there is more than one group | *7-10* | **Variables**  **Outcome variables**  The primary outcome was the proportion of participants with at least one atypical breast. Participants were asked to indicate what each of their breasts individually looked like just prior to pregnancy with their first child using Figure in S2 Fig. Participants who had breast surgery prior to the birth of their first child were asked to report breast appearance prior to surgery.  A secondary outcome was the feasibility of self-administration of the survey based on the proportion for whom breast type and other individual markers suggestive of breast hypoplasia could be determined. Additional secondary outcomes included the proportion of participants with other individual markers suggestive of breast hypoplasia as well as a delay in secretory activation. We also estimated the odds of having markers of breast hypoplasia in at-risk groups compared to reference groups, adjusting for covariates.  **Exposure variables**  Various endocrine alterations including polycystic ovary syndrome (PCOS), diabetes (type I, II or gestational) and hypothyroidism have been identified as being associated with breast hypoplasia [8-17]. Therefore, participants were asked whether they had any such endocrine conditions medically diagnosed prior to the birth of their first child. Data were collected about the timing of onset of these conditions and medications used to manage a diagnosis of PCOS, gestational diabetes mellitus (GDM) or type II diabetes. Participants were asked to provide estimates of their height and weight just before their index pregnancy in order to calculate their pre-pregnancy BMI. BMI was defined as per the World Health Organization BMI categories [31]: <18.5 kg/m^2^ (underweight), 18.5 to <25.0 kg/m^2^ (normal weight), 25.0 to <30.0 kg/m^2^ (overweight), 30.0 to <35.0 kg/m^2^ (obese class 1), 35.0 to <40.0 kg/m^2^ (obese class 2), and ≥40.0 kg/m^2^ (obese class 3).  **Other covariates**  Demographic characteristics including current age, country of residence, marital status and education were collected. Ethnicity was collected separately for each country where eligible women usually resided in. For participants who typically resided in Australia, questions related to indigeneity and country of birth were asked. Data about intention to breastfeed (by asking how long women planned to breastfeed their baby for) were collected.  Participants were also asked about conditions, obstetric history (method of birth and analgesia used during labour) or surgery which may interfere with mammary glandular tissue development and/or lactation capacity. A final open-ended question was asked about participants’ personal stories of how their low milk supply was discovered or diagnosed (not included in this paper). |
| Bias | 9 | Describe any efforts to address potential sources of bias |  | An eligibility check was undertaken prior to participants undertaking the survey. Recall bias was attempted to be minimised by participants being required to have had their baby within the previous 5 years. Covariates were adjusted for in logistic regression analyses. The study limitations are made clear in the discussion section. |
| Study size | 10 | Explain how the study size was arrived at | 12 | **Sample size**  Sample size was calculated to estimate the proportion of participants with at least one atypical breast [32]. *A priori,* we estimated the proportion of women with at least one atypical breast to be 50%. To ensure the 95% confidence interval (CI) estimate of the proportion of women who report low milk supply with at least one atypical breast is within 5% of the true population proportion, a sample of 385 was needed. Accounting for a 20% incomplete survey response, we aimed to recruit 482 women. |

Continued on next page

| Quantitative variables | 11 | Explain how quantitative variables were handled in the analyses. If applicable, describe which groupings were chosen and why | 12-13 | **Statistical analyses**  **Primary outcome**  The estimated proportion of women in our sample having at least one atypical breast and the 95% CI around the estimate was determined. The numerator was based on the total number of participants coded ‘atypical’ and the denominator represented the sum of participants coded as ‘typical’ plus ‘atypical’ based on their responses. “None”, “unsure” and missing responses were excluded from the primary result; in sensitivity analysis, we included these responses in the denominator to determine the potential impact of their missingness on estimated prevalence of atypical breast in this population.  **Secondary outcomes**  The feasibility of collecting information directly from women using an online survey was measured by calculating the proportion of respondents definitively answering the items related to the primary and secondary outcomes, compared to the proportion who skipped answering these items or indicated ‘unsure.’ Participants’ open text responses were examined to identify any indication of confusion or feedback about these items.  The proportion of participants with proposed markers of breast hypoplasia was estimated and 95% CI calculated. “Unsure” and missing responses were not included in these analyses.  The Chi-square (χ2) test was used to examine bivariate associations between exposure and outcome variables. Effect sizes were determined using Cramer’s V. For associations where p<0.10, multiple logistic regression was used to estimate the odds of the outcome in the at-risk group compared to the reference group, adjusting for covariates.  We performed all statistical analyses in Stata version 15 [33]. The significance level used was p<0.05. Reporting for this study followed the Checklist for Reporting Results of Internet E-Surveys (CHERRIES) statement (File in S3 File) [26]. |
| --- | --- | --- | --- | --- |
| Statistical methods | 12 | (*a*) Describe all statistical methods, including those used to control for confounding | 12-13 | **Statistical analyses**  **Primary outcome**  The estimated proportion of women in our sample having at least one atypical breast and the 95% CI around the estimate was determined. The numerator was based on the total number of participants coded ‘atypical’ and the denominator represented the sum of participants coded as ‘typical’ plus ‘atypical’ based on their responses. “None”, “unsure” and missing responses were excluded from the primary result; in sensitivity analysis, we included these responses in the denominator to determine the potential impact of their missingness on estimated prevalence of atypical breast in this population.  **Secondary outcomes**  The feasibility of collecting information directly from women using an online survey was measured by calculating the proportion of respondents definitively answering the items related to the primary and secondary outcomes, compared to the proportion who skipped answering these items or indicated ‘unsure.’ Participants’ open text responses were examined to identify any indication of confusion or feedback about these items.  The proportion of participants with proposed markers of breast hypoplasia was estimated and 95% CI calculated. “Unsure” and missing responses were not included in these analyses.  The Chi-square (χ2) test was used to examine bivariate associations between exposure and outcome variables. Effect sizes were determined using Cramer’s V. For associations where p<0.10, multiple logistic regression was used to estimate the odds of the outcome in the at-risk group compared to the reference group, adjusting for covariates.  We performed all statistical analyses in Stata version 15 [33]. The significance level used was p<0.05. Reporting for this study followed the Checklist for Reporting Results of Internet E-Surveys (CHERRIES) statement (File in S3 File) [26]. |
|  |  | (*b*) Describe any methods used to examine subgroups and interactions | N/A |  |
|  |  | (*c*) Explain how missing data were addressed | 12-13 | **Statistical analyses**  **Primary outcome**  The estimated proportion of women in our sample having at least one atypical breast and the 95% CI around the estimate was determined. The numerator was based on the total number of participants coded ‘atypical’ and the denominator represented the sum of participants coded as ‘typical’ plus ‘atypical’ based on their responses. “None”, “unsure” and missing responses were excluded from the primary result; in sensitivity analysis, we included these responses in the denominator to determine the potential impact of their missingness on estimated prevalence of atypical breast in this population.  **Secondary outcomes**  The feasibility of collecting information directly from women using an online survey was measured by calculating the proportion of respondents definitively answering the items related to the primary and secondary outcomes, compared to the proportion who skipped answering these items or indicated ‘unsure.’ Participants’ open text responses were examined to identify any indication of confusion or feedback about these items.  The proportion of participants with proposed markers of breast hypoplasia was estimated and 95% CI calculated. “Unsure” and missing responses were not included in these analyses.  The Chi-square (χ2) test was used to examine bivariate associations between exposure and outcome variables. Effect sizes were determined using Cramer’s V. For associations where p<0.10, multiple logistic regression was used to estimate the odds of the outcome in the at-risk group compared to the reference group, adjusting for covariates.  We performed all statistical analyses in Stata version 15 [33]. The significance level used was p<0.05. Reporting for this study followed the Checklist for Reporting Results of Internet E-Surveys (CHERRIES) statement (File in S3 File) [26]. |
|  |  | (*d*) *Cohort study*—If applicable, explain how loss to follow-up was addressed  *Case-control study*—If applicable, explain how matching of cases and controls was addressed  *Cross-sectional study*—If applicable, describe analytical methods taking account of sampling strategy | N/A |  |
|  |  | (*e*) Describe any sensitivity analyses | N/A |  |
| Results | | | | |
| Participants | 13* | (a) Report numbers of individuals at each stage of study—eg numbers potentially eligible, examined for eligibility, confirmed eligible, included in the study, completing follow-up, and analysed | 13,16 | A total of 487 participants commenced the survey; 399 responded to our primary outcome (breast type) (81.9%).  Survey participants were able to comprehend the survey items about markers suggestive of breast hypoplasia, with over 80% responding to these items (Table in S2 Table). |
|  |  | (b) Give reasons for non-participation at each stage | N/A |  |
|  |  | (c) Consider use of a flow diagram |  |  |
| Descriptive data | 14* | (a) Give characteristics of study participants (eg demographic, clinical, social) and information on exposures and potential confounders | 13-14, table 1 | Of participants who responded to the breast type question, 67.9% resided in the United States of America, 23.3% in Australia and 8.8% in the United Kingdom (Table 1). The mean age of participants was 32 years (SD 4.6) and most (84%) had either a bachelor or postgraduate degree. The majority (85.1%) of participants intended to breastfeed for at least 12 months (320/376). Socio-demographic characteristics based on data from participants who responded to the breast type question are summarised in Table 1. |
|  |  | (b) Indicate number of participants with missing data for each variable of interest | S2 Table | Please refer to S2 table |
|  |  | (c) *Cohort study*—Summarise follow-up time (eg, average and total amount) | N/A |  |
| Outcome data | 15* | *Cohort study*—Report numbers of outcome events or summary measures over time | N/A |  |
|  |  | *Case-control study—*Report numbers in each exposure category, or summary measures of exposure | N/A |  |
|  |  | *Cross-sectional study—*Report numbers of outcome events or summary measures | N/A |  |
| Main results | 16 | (*a*) Give unadjusted estimates and, if applicable, confounder-adjusted estimates and their precision (eg, 95% confidence interval). Make clear which confounders were adjusted for and why they were included | 14-23, tables 2-5 and S3 Table | One sample proportion tests with CIs and chi-square analyses were performed. In addition, adjusted and unadjusted logistic regression results are presented. Please refer to results section for details.  Here is the relevant text for the logistic regression analyses:  We performed nine separate multiple logistic regression analyses on the relationships for which Chi-square analyses had a p value <0.1. These relationships included between: i) BMI and atypical breast type, ii) BMI and widely spaced breasts, iii) BMI and stretch marks on the breast, iv) BMI and lack of pregnancy breast growth; v) PCOS and atypical breast type, vi) PCOS and stretch marks on the breast, vii) widely spaced breasts and atypical breast type, viii) lack of pregnancy breast growth and atypical breast type, and ix) GDM and lack of pregnancy breast growth. Crude and adjusted odds ratios were obtained for these relationships by performing bivariate logistic regression analyses and multiple logistic regression analyses adjusting for covariates.  Various relationships between BMI and proposed markers of breast hypoplasia remained significant in multiple logistic regression models after adjusting for covariates (age, country of residence, PCOS, GDM and hypothyroidism) (Table in S3 Table). The normal weight category was used as the reference category. In the adjusted model, the odds of having widely spaced breasts were 1.97 (95% CI: 1.17, 3.32) times and 2.21 (95% CI: 1.26, 3.86) higher among women in the overweight and obese 1 category, respectively, compared to women with normal weight. The odds of having stretch marks on the breast were 3.97 (95% CI: 1.55, 10.14) times higher among women in the obese 1 category compared to women with normal weight. The odds of a lack of pregnancy breast growth were also significantly more likely among women in the overweight (2.27 [95% CI: 1.29, 4.02]), obese 1 (2.07 [95% CI: 1.12, 3.80]) and obese 2+ (3.52 [95% CI: 1.63, 7.58]) categories. The relationship between BMI and atypical breast type was no longer significant in the adjusted model.  When adjusted for BMI, country of residence and age, multiple logistic regression analyses revealed no evidence of an association between PCOS and the presence of stretch marks on the breast (1.79 [95% CI: 0.72, 4.42]) nor between GDM and lack of pregnancy growth (1.61 [95% CI: 0.74, 3.54]) and some evidence of a relationship between PCOS and atypical breast type (1.94 [95% CI: 0.99, 3.77]) (data not shown).  We used multiple logistic regression analyses to investigate whether women with atypical breasts might be more likely to have other proposed breast hypoplasia markers. When adjusted for BMI, PCOS, GDM, hypothyroidism, age and country of residence, the odds of women having at least one atypical breast was 8.86 times higher in women with widely spaced breasts and 3.39 times higher in women with a lack of pregnancy breast growth compared to women without at least one atypical breast (95% CI: 4.88, 16.07; 95% CI: 2.01, 5.71) (data not shown). |
|  |  | (*b*) Report category boundaries when continuous variables were categorized | N/A |  |
|  |  | (*c*) If relevant, consider translating estimates of relative risk into absolute risk for a meaningful time period | N/A |  |

Continued on next page

| Other analyses | 17 | Report other analyses done—eg analyses of subgroups and interactions, and sensitivity analyses | N/A |  |
| --- | --- | --- | --- | --- |
| Discussion | | | | |
| Key results | 18 | Summarise key results with reference to study objectives | 23-25 | To our knowledge, this is the first study reporting the proportion of anatomical breast characteristics among women from three countries who self-report insufficient milk supply. In our sample, 68% of women reported having at least one atypical breast (i.e., a type 2, 3 or 4 breast per Fig 1 in Huggins et al [21]). Over 80% of our sample responded to the breast anatomy survey questions demonstrating it is feasible to ask women to self-report markers suggestive of breast hypoplasia, and providing confidence about the content validity of our findings.  Lack of pregnancy breast growth, breast asymmetry and the presence of stretch marks on the breast have been identified as potential markers of breast hypoplasia [5]. Over three-quarters (76%) of women in our sample reported a lack of pregnancy breast growth. This figure is considerably higher than 24% (75/319) and 18% (35/192) reporting this phenomenon among healthy breastfeeding primiparous women and primiparous (42%) and multiparous (28%) women with a BMI <27, respectively, who gave birth to healthy term newborns [20,35]. Also, in a socioeconomic diverse cohort of primiparous women, 7% (30/431) reported no prenatal breast enlargement [25]. The difference in these rates may be explained by our sample being women reporting low milk supply. Breast asymmetry was examined antenatally by Neifert et al who found that 8% (24/319) had ‘moderate’ and 0.3% (1/319) ‘marked’ asymmetry (no further detail is provided about these descriptions) [20]. Similarly, in our sample, 8% of women reported a ≥ 2 cup size difference between their breasts. As reported by Picard and colleagues, the breasts of 800 consecutive women (with a mean BMI of 23 and mean age of 26 years) were examined by the same dermatologist and the prevalence of breast stretch marks was 33% [36]. Obesity, higher pre-pregnancy BMI and higher gestational weight gain have been identified as risk factors for the development of stretch marks in pregnancy [36,37]. In our sample, 72% of women reported the presence of stretch marks on their breasts prior to the birth of their first child.  Obesity remains a significant public health concern in high and middle income countries with data showing the prevalence of obesity among reproductive age women to be over 40% in the USA and 30% in Australia and England [38-40]. Obesity is common among women self-reporting low milk supply and has been linked to decreased breastfeeding initiation, shorter breastfeeding duration, lower milk supply and delayed secretory activation [41,42]. It is possible that endocrine disturbances associated with high BMI might alter breast development [43]. Vanky et al’s study of 186 women with PCOS found those with no increase in bra size during pregnancy had larger BMIs compared with those who experienced breast size increment [8]. Comparably, in our study, adjusted multiple logistic regression analyses found associations between high BMI and widely spaced breasts, lack of pregnancy breast growth and stretch marks.  We investigated whether a relationship exists between atypical breast type and other proposed markers of breast hypoplasia and found evidence of a link between atypical breast type and both lack of pregnancy breast growth and widely spaced breasts. This supports the findings by Huggins et al who found that among women with type 2, 3 or 4 breasts, 76% (22/29) and 86% (25/29) also had minimal or no pregnancy breast growth and widely spaced breasts respectively [21]. |
| Limitations | 19 | Discuss limitations of the study, taking into account sources of potential bias or imprecision. Discuss both direction and magnitude of any potential bias | 25-26 | **Limitations**  There are several limitations of this study. We used a convenience sample of women who were members of low milk supply online support groups. The sample was self-selected and biased to well-educated mothers, with a high breastfeeding intention. The reasons for the women’s low milk supply are unknown. All exposure and outcome variables were identified via self-report and therefore lack objectivity, and we recognise that recall and confirmation biases are possible. The survey was accessed via Facebook, limiting access to women without the internet or social media accounts. The high proportion of women in this sample with various proposed breast hypoplasia markers does not imply that these factors are ‘risks’ for low milk supply since the proportion of women with normal milk production/general population with these markers is unknown. |
| Interpretation | 20 | Give a cautious overall interpretation of results considering objectives, limitations, multiplicity of analyses, results from similar studies, and other relevant evidence | 26-27 | Members of Facebook groups for women with low milk supply have had high rates of atypical breasts (at least one breast being type 2, 3 or 4 as per Fig 1 in Huggins et al [21]), and often reported no breast growth in pregnancy. Women with larger bodies were more likely to have a number of features of breast hypoplasia including widely spaced breasts, stretch marks on the breast and lack of pregnancy breast growth. To ascertain the strongest set of breast hypoplasia markers for predicting low supply, these findings must be confirmed in large well-designed cohort studies. Fundamental to helping more women to make a full milk supply to enable exclusive breastfeeding is an understanding that breastfeeding is a physiological function that promotes maternal physical and mental health [47]. When women encounter difficulty conceiving, they seek to understand why and treatment to help. Likewise, women unable to make a full milk supply also deserve to have their challenges investigated and explained. Therefore, it is time that human lactation became a research priority. |
| Generalisability | 21 | Discuss the generalisability (external validity) of the study results | 25 | We used a convenience sample of women who were members of low milk supply online support groups. The sample was self-selected and biased to well-educated mothers, with a high breastfeeding intention. The reasons for the women’s low milk supply are unknown. All exposure and outcome variables were identified via self-report and therefore lack objectivity, and we recognise that recall and confirmation biases are possible. The survey was accessed via Facebook, limiting access to women without the internet or social media accounts. The high proportion of women in this sample with various proposed breast hypoplasia markers does not imply that these factors are ‘risks’ for low milk supply since the proportion of women with normal milk production/general population with these markers is unknown. |
| Other information | |  | | |
| Funding | 22 | Give the source of funding and the role of the funders for the present study and, if applicable, for the original study on which the present article is based | N/A |  |

*Give information separately for cases and controls in case-control studies and, if applicable, for exposed and unexposed groups in cohort and cross-sectional studies.

**Note:** An Explanation and Elaboration article discusses each checklist item and gives methodological background and published examples of transparent reporting. The STROBE checklist is best used in conjunction with this article (freely available on the Web sites of PLoS Medicine at http://www.plosmedicine.org/, Annals of Internal Medicine at http://www.annals.org/, and Epidemiology at http://www.epidem.com/). Information on the STROBE Initiative is available at www.strobe-statement.org.
