# Supplementary figures and images for: Breast hypoplasia markers among women who report insufficient milk production: A retrospective online survey
Source: PLoS One. 2024 Feb 29;19(2):e0299642. doi: 10.1371/journal.pone.0299642 (PMC10903845; doi:10.1371/journal.pone.0299642)

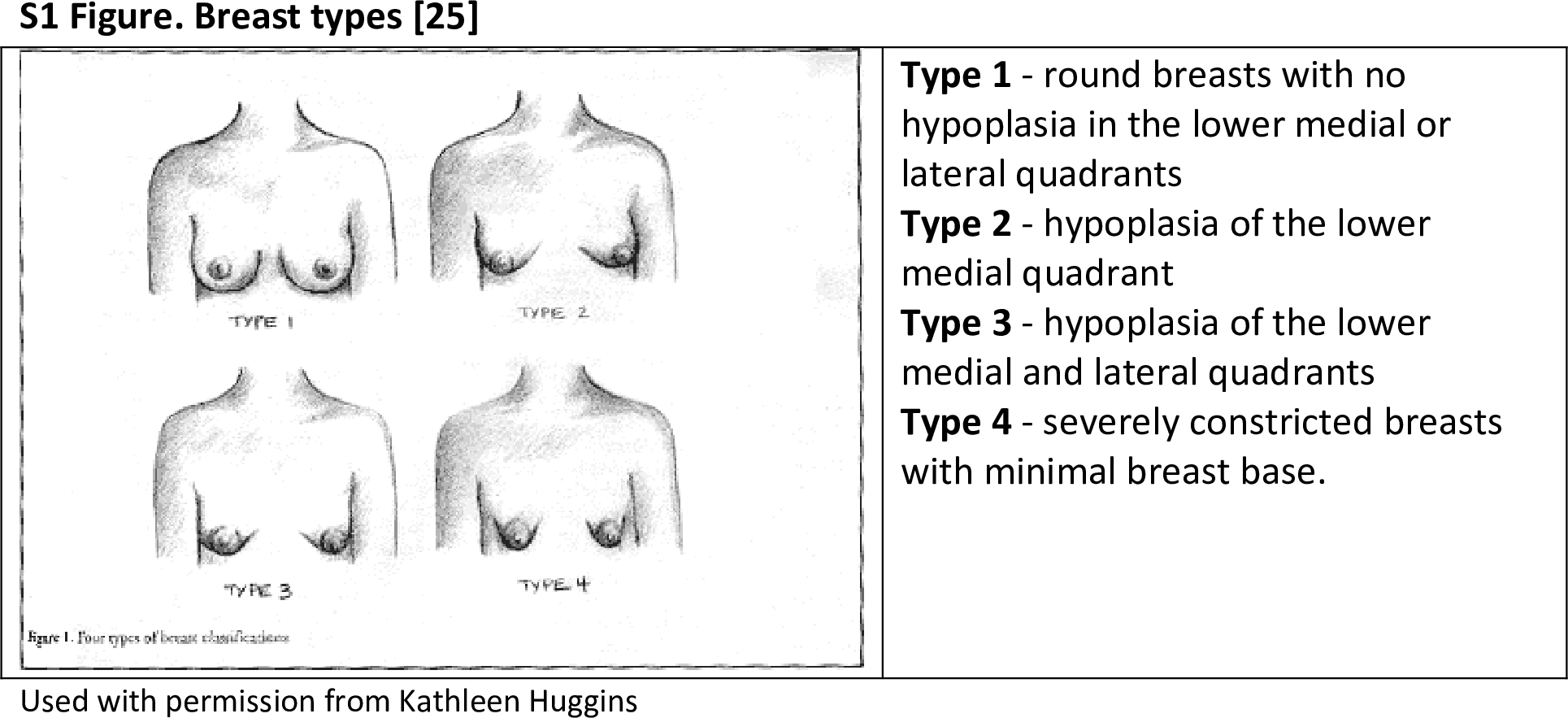

Supplement: S1 Fig — (TIF) [file pone.0299642.s002.tif]

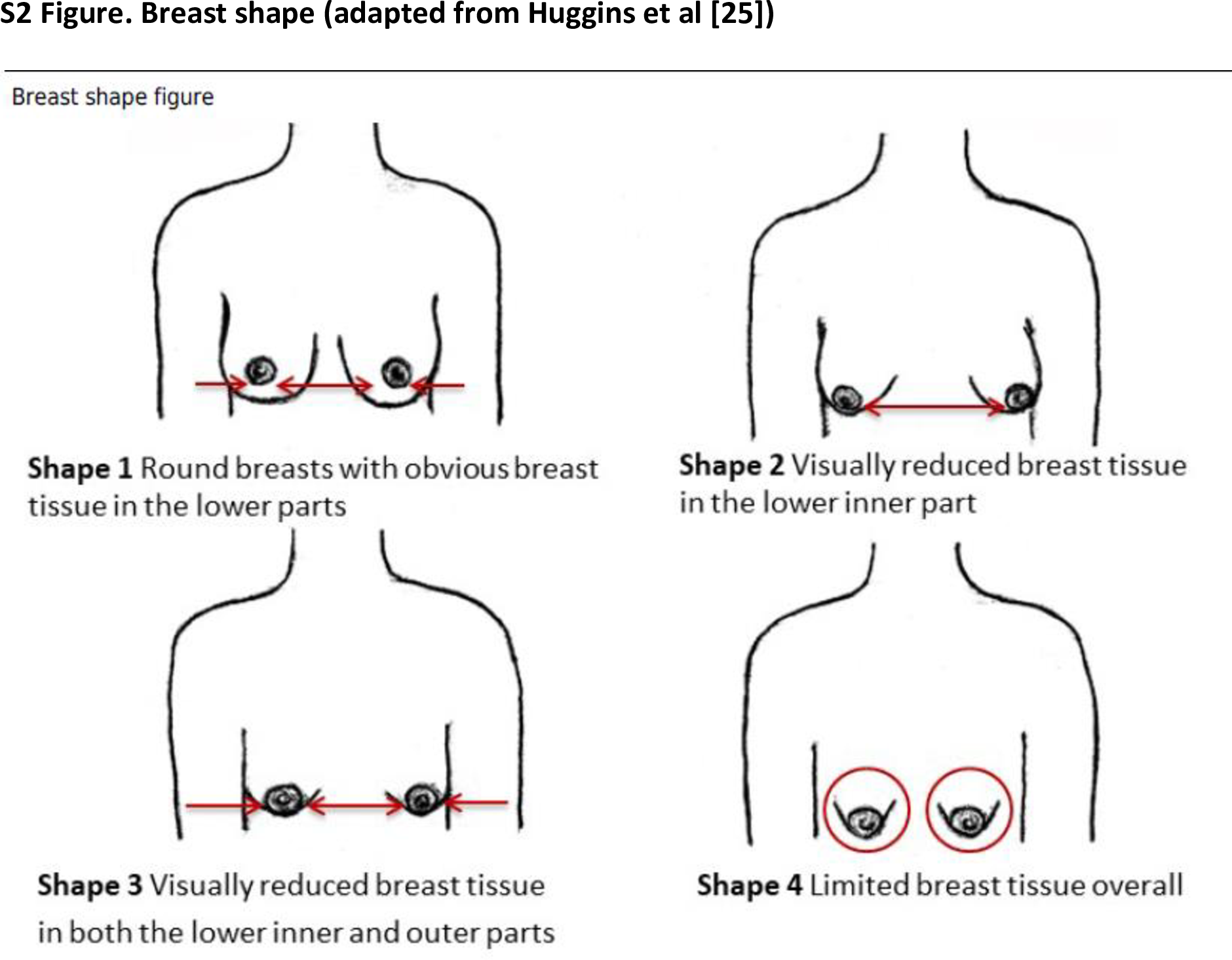

Supplement: S2 Fig — (TIF) [file pone.0299642.s003.tif]
