# Supplementary material for: Breast hypoplasia markers among women who report insufficient milk production: A retrospective online survey
Source: PLoS One. 2024 Feb 29;19(2):e0299642. doi: 10.1371/journal.pone.0299642 (PMC10903845; doi:10.1371/journal.pone.0299642)
Supplement: S1 Table — (PDF) [file pone.0299642.s004.pdf]

S1 Table. Facebook groups where invitations were posted

Name/description	Number of members*	Explanation of group	Type of group or page‡
IGT And Low Milk Supply Support Group (Administrators from the USA)	10,687	Targeted towards subset of parents who are unable to make sufficient breastmilk	Private group
Supply Line Breastfeeders Support Group of Australia (Administrators from Australia)	2,737	Aims to provide support for the use of at-breast supplementation devices (supply lines)	Private group
IGT Off Topic Group (Administrators from the USA)	2,378	Must be a member of the 'IGT And Low Milk Supply Support Group' group to join. Discussion about broader topics than just breastfeeding issues	Private group
Low Milk Supply - A Mother's Love (Administrators from the USA)	2,594	For any mother who cannot produce enough to feed her baby	Private group
Low Milk Supply/Domperidone (Administrators from the USA and Sri Lanka)	2,716	For information about domperidone	Private group
First author's personal profile (Owner from Australia)	N/A		Personal profile
First author's lactation consultancy business page (Owner from Australia)	Followed by 300 people		Business page

*As of 20th August 2022

‡In private Facebook groups, only members can see who is in the group and what they post.

A Facebook profile is where an individual can share information about themselves. The profile owner chooses what audience sees each post they create.

Facebook pages are usually created to represent a business, organisation or public person. Pages are always public, meaning anyone can search for, and view the page's posts.

IGT, insufficient glandular tissue; USA, United States of America
