# Supplementary material for: Breast hypoplasia markers among women who report insufficient milk production: A retrospective online survey
Source: PLoS One. 2024 Feb 29;19(2):e0299642. doi: 10.1371/journal.pone.0299642 (PMC10903845; doi:10.1371/journal.pone.0299642)
Supplement: S2 Table — (PDF) [file pone.0299642.s005.pdf]

S2 Table. Proportion of participants who answered questions about markers suggestive of breast hypoplasia

Breast hypoplasia marker the survey question pertained to	n/N (%)
Breast type (right)*	399/487 (81.9)
Breast type (left)*	399/487 (81.9)
Intermammary width	449/487 (92.2)
Breast asymmetry	448/487 (92.0)
Breast growth in pregnancy	449/487 (92.2)
Presence of stretch marks on one or both breasts	452/487 (92.8)
Stretch marks (appeared before birth of first child)	250/293 (85.3)
Stretch marks (appeared between 8 and 20 years of age)	168/191 (88.0)
Stretch marks (appeared during pregnancy with first child)	186/191 (97.4)
Timing of symptoms of secretory activation	439/487 (90.1)

*Includes 51 responses missing due to branching logic error in REDCap survey
