# Supplementary material for: Breast hypoplasia markers among women who report insufficient milk production: A retrospective online survey
Source: PLoS One. 2024 Feb 29;19(2):e0299642. doi: 10.1371/journal.pone.0299642 (PMC10903845; doi:10.1371/journal.pone.0299642)
Supplement: S3 Table — (PDF) [file pone.0299642.s006.pdf]

S3 Table. Association of BMI with proposed markers of breast hypoplasia

	Breast anatomy characteristic*									
	>=1 atypical		Widely spaced		Asymmetry		Stretch marks		Lack of growth	
	Crude OR (95% CI) (N=385)	Adjusted OR (95% CI) (N=365)	Crude OR (95% CI) (N=385)	Adjusted OR (95% CI) (N=365)	Crude OR (95% CI) (N=385)	Adjusted OR (95% CI) (N=365)	Crude OR (95% CI) (N=385)	Adjusted OR (95% CI) (N=365)	Crude OR (95% CI) (N=385)	Adjusted OR (95% CI) (N=365)
Normal weight (ref.cat)	1	1	1	1	1	1	1	1	1	1
Overweight	1.78	1.63	**2.13**	**1.97**	1.60	1.80	1.63	1.53	**2.43**	**2.27**
	(1.02, 3.12)‡	(0.90, 2.94)	**(1.30, 3.48)‡‡**	**(1.17, 3.32)‡**	(0.66, 3.91)	(0.70, 4.63)	(0.76, 3.50)	(0.69, 3.37)	**(1.41, 4.18)‡‡**	**(1.29, 4.02)‡**
Obese class 1	**2.15**	1.85	**2.49**	**2.21**	1.17	1.22	**3.98**	**3.97**	**2.17**	**2.07**
	**(1.17, 3.97)‡**	(0.97, 3.51)	**(1.46, 4.24)‡‡**	**(1.26, 3.86)‡‡**	(0.41, 3.34)	(0.41, 3.68)	**(1.60, 9.88)‡‡**	**(1.55, 10.14)‡‡**	**(1.21, 3.88)‡‡**	**(1.12, 3.80)‡**
Obese class 2+	1.74	1.27	**1.86**	1.27	1.57	1.78	**2.72**	2.55	**3.96**	**3.52**
	(0.92, 3.29)	(0.62, 2.60)	**(1.06, 3.26)‡**	(0.68, 2.36)	(0.57, 4.29)	(0.60, 5.33)	**(1.15, 6.44)‡**	(0.996, 6.51)	**(1.94, 8.06)‡‡‡**	**(1.63, 7.58)‡‡**

*>=1 atypical, at least one breast atypical; Widely spaced, intermammary width > 1.5 inches or 3.8 cm; Asymmetry, ≥ 2 cup size difference between breasts; Stretch marks, stretch marks on one or both breast/s prior to index child; Lack of growth, lack of breast growth during pregnancy defined as no noticeable change in or an increase of < 1 bra cup size to either breast during index pregnancy

ƚnormal weight, 18.5 to <25.0 kg/m^2; overweight, 25.0 to <30.0 kg/m^2; obese class 1, 30.0 to <35.0 kg/m^2; obese class 2+, ≥35.0 kg/m^2. Underweight category excluded due to inadequate sample size (n=5). Obese categories 2 and above combined due to small sample sizes (n=39 for obese 2 and n=35 for obese3).

‡p<0.05 ‡‡p<0.01 ‡‡‡p<0.001

¨Adjusted for age, country of residence, PCOS (polycystic ovary syndrome), GDM (gestational diabetes mellitus) and hypothyroidism

ref cat, reference category
