# Supplementary material for: Breast hypoplasia markers among women who report insufficient milk production: A retrospective online survey
Source: PLoS One. 2024 Feb 29;19(2):e0299642. doi: 10.1371/journal.pone.0299642 (PMC10903845; doi:10.1371/journal.pone.0299642)
Supplement: S4 Table — (DOCX) [file pone.0299642.s007.docx]

**S4 Table. Logistic regression modelling of risk factors for widely spaced breasts**

| **Metabolic characteristic** | **Reference category** | **Crude OR (95% CI)** | **Model 1 AOR**^*^ **(95% CI)** | **Model 2 AOR^ƚ^ (95% CI)** |
| --- | --- | --- | --- | --- |
| **BMI**^ǂ^ **(kg/m^2^)** | BMI 18.5 to ≤25.0 |  |  |  |
| 25.0 to <30.0 |  | **2.13**  **(1.30, 3.48)^ǂǂ^** | 1.61  (0.89, 2.92) | 1.24  (0.63, 2.43) |
| 30.0 to <35.0 |  | **2.49**  **(1.46, 4.24)^ǂǂ^** | **2.45**  **(1.25, 4.79)^ǂǂ^** | 1.75  (0.76, 4.01) |
| ≥35.0 |  | **1.86**  **(1.06, 3.26)^ǂ^** | 1.67  (0.86, 3.24) | 1.01  (0.44, 2.33) |
| **Youth weight**^-^ | Normal weight |  |  |  |
| A little overweight |  | **1.72**  **(1.04, 2.87)^ǂ^** | -- | 0.96  (0.49, 1.86) |
| Moderately / very overweight |  | **2.57**  **(1.52, 4.33)^ǂǂǂ^** | -- | 1.89  (0.90, 3.96) |

^*^Adjusted for country of residence and USA ethnicity

**^ƚ^**Adjusted for all in model 1 plus youth weight category

^ǂ^Underweight category excluded due to inadequate sample size (n=5).

^-^Youth weight, description of weight between 8 and 20 years of age. Underweight excluded due to inadequate sample size (n=16). Moderately and very overweight categories combined (n=76 for moderately overweight and n=32 for very overweight)

^ǂ^p<0.05, ^ǂǂ^p≤0.01, ^ǂǂǂ^p≤0.001

BMI, body mass index
