# Supplementary material for: Breast hypoplasia markers among women who report insufficient milk production: A retrospective online survey
Source: PLoS One. 2024 Feb 29;19(2):e0299642. doi: 10.1371/journal.pone.0299642 (PMC10903845; doi:10.1371/journal.pone.0299642)
Supplement: S5 Table — (DOCX) [file pone.0299642.s008.docx]

**S5 Table. Logistic regression modelling of risk factors for presence of stretch marks prior to birth of first child**

| **Metabolic characteristic** | **Reference category** | **Crude OR (95% CI)** | **Model 1 AOR**^*^ **(95% CI)** |
| --- | --- | --- | --- |
| **PCOS** | No PCOS | **2.57 (1.09, 6.06)^ǂ^** | **3.17 (1.02, 9.81)^ǂ^** |
| **BMI^ƚ^ (kg/m^2^)** | BMI 18.5 to ≤25.0 |  |  |
| 25.0 to <30.0 |  | 1.63  (0.76, 3.50) | 1.09  (0.43, 2.76) |
| 30.0 to <35.0 |  | **3.98**  **(1.60, 9.88)^ǂǂ^** | 2.52  (0.82, 7.72) |
| ≥35.0 |  | **2.72**  **(1.15, 6.44)^ǂ^** | 1.57  (0.55, 4.47) |

^*^Adjusted for USA ethnicity, PCOS and BMI

**^ƚ^**Underweight category excluded due to inadequate sample size (n=5).

BMI, body mass index; PCOS, polycystic ovary syndrome
