# Supplementary material for: Breast hypoplasia markers among women who report insufficient milk production: A retrospective online survey
Source: PLoS One. 2024 Feb 29;19(2):e0299642. doi: 10.1371/journal.pone.0299642 (PMC10903845; doi:10.1371/journal.pone.0299642)
Supplement: S6 Table — (DOCX) [file pone.0299642.s009.docx]

**S6 Table. Logistic regression modelling of risk factors for presence of lack of breast growth in pregnancy with first child**

| **Metabolic characteristic** | **Reference category** | **Crude OR (95% CI)** | **Model 1 AOR**^*^ **(95% CI)** | **Model 2 AOR ^ƚ^ (95% CI)** |
| --- | --- | --- | --- | --- |
| **GDM** | No GDM | 2.03  (0.96, 4.29) | 1.61  (0.74, 3.54) | 1.46  (0.66, 3.25) |
| **BMI**^ǂ^ **(kg/m^2^)** | BMI 18.5 to ≤25.0 |  |  |  |
| 25.0 to <30.0 |  | **2.43**  **(1.41, 4.18)^ǂǂ^** | **2.20**  **(1.25, 3.86)^ǂǂ^** | 1.57  (0.85, 2.91) |
| 30.0 to <35.0 |  | **2.17**  **(1.21, 3.88)^ǂǂ^** | **1.88**  **(1.04, 3.41)^ǂ^** | 1.28  (0.63, 2.62) |
| ≥35.0 |  | **3.96**  **(1.94, 8.06)^ǂǂǂ^** | **3.00**  **(1.44, 6.28)^ǂǂ^** | 1.73  (0.74, 4.07) |
| **Youth weight**^-^ | Normal weight |  |  |  |
| A little overweight |  | **2.05**  **(1.22, 3.45)^ǂǂ^** | -- | 1.54  (0.86, 2.75) |
| Moderately / very overweight |  | **3.21**  **(1.80, 5.70)^ǂǂǂ^** | -- | **2.35**  **(1.16, 4.78)^ǂ^** |

^*^Adjusted for age, country of residence, GDM and BMI

**^ƚ^**Adjusted for all in model 1 plus youth weight category

^ǂ^Underweight category excluded due to inadequate sample size (n=5).

^-^Youth weight, description of weight between 8 and 20 years of age. Underweight excluded due to inadequate sample size (n=16). Moderately and very overweight categories combined (n=76 for moderately overweight and n=32 for very overweight)

^ǂ^p<0.05, ^ǂǂ^p≤0.01, ^ǂǂǂ^p≤0.001

GDM, gestational diabetes mellitus; BMI, body mass index
