# Supplementary material for: Breast hypoplasia markers among women who report insufficient milk production: A retrospective online survey
Source: PLoS One. 2024 Feb 29;19(2):e0299642. doi: 10.1371/journal.pone.0299642 (PMC10903845; doi:10.1371/journal.pone.0299642)
Supplement: S1 File — (PDF) [file pone.0299642.s010.pdf]

Survey - Breast Variations Among Women With Low Milk Supply

Thank you again for your interest in this study. Again, the survey is anonymous – we will not be able to identify you. It will take 15-20 minutes to complete. If you cannot finish the survey in one go, you can save it and return to it later.

If you have more than one child, please answer the questions based on your first child (i.e., your oldest child) who was a live birth.

Australian English is the language which was chosen to write this survey. Therefore, for responders from the USA, the spelling of some words may differ from what you're used to (e.g., 'caesarean' rather than 'cesarean').

There are no right or wrong answers to any of the questions in this survey. We are interested in your answers. If there are any questions you would prefer not to answer, simply move to the next question.

If any of the questions in this survey leave you with questions or concerns about you or your baby please contact a member of your healthcare team. This might include your GP or primary physician. For support around any aspect of breastfeeding, you might like to consider phoning the:

National Breastfeeding Helpline on 1800 686 268 if you live in AustraliaNational Women's Health and Breastfeeding Helpline on 1 800 994 9662 if you live in the United States National Breastfeeding Helpline UK on 0300 100 0212 if you live in the United Kingdom

We would like to start by asking you some questions about yourself.

In which country do you usually reside?

- ◯ United States of America
- ◯ Australia
- ◯ United Kingdom

In which country were you born?

- ◯ Australia
- ◯ England
- ◯ New Zealand
- ◯ India
- ◯ Philippines
- ◯ Vietnam
- ◯ Italy
- ◯ South Africa
- ◯ Malaysia
- ◯ Scotland
- ◯ Other

What 'other' country were you born in?

____

Are you of Aboriginal or Torres Strait Islander origin?

- ◯ Yes
- ◯ No

Which ethnic background do you identify with? (Tick all that apply).

- ☐ White
- ☐ Black, African, Caribbean or Black British
- ☐ Asian or Asian British
- ☐ Other

What 'other' ethnic group do you identify with?

____

Which ethnic background do you identify with? (Tick all that apply).

- ☐ White
- ☐ Black, African or Caribbean
- ☐ Asian
- ☐ Hispanic/Latina
- ☐ Other

What 'other' ethnic group do you identify with?	__________________________________
What is your age in years?	__________________________________
When your first child was born, what was your marital status?	◯ Single<br>◯ Married or living with partner<br>◯ Widowed<br>◯ Separated<br>◯ In a relationship but not living together<br>◯ Other
What 'other' marital status did you have when your first child was born?	__________________________________
What is the highest level of education you've attained?	◯ Primary school<br>◯ Secondary school/high school<br>◯ Some training beyond secondary school or high school<br>◯ Bachelor degree<br>◯ Postgraduate degree<br>◯ Other
What 'other' highest education level have you attained?	__________________________________

Now we'd like to ask you some questions about your breasts.

What is the cup size difference between each of your breasts today? You can make a guess

- ◯ 0 cup size difference
- ◯ 1 cup size difference
- ◯ 2 cup size difference
- ◯ 3 cup size difference
- ◯ 4 or more cup size difference
- ◯ Unsure

Please comment about why you're unsure of the cup size difference between your breasts.

____

Do you have any stretch marks on one or both of your breasts?

- ◯ No
- ◯ Yes
- ◯ Unsure
- ◯ Prefer not to say

Did the stretch marks first appear before the birth of your first child?

- ◯ No
- ◯ Yes
- ◯ Unsure

Did the stretch marks first develop between 8 and 20 years of age?

- ◯ No
- ◯ Yes
- ◯ Unsure

Did the stretch marks first develop during pregnancy with your first child?

- ◯ No
- ◯ Yes
- ◯ Unsure

Width of space between breasts

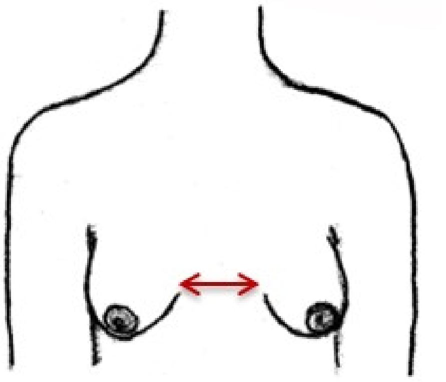

Please refer to image just viewed. Thinking about the shortest distance between where your breasts attach to your chest wall, how widely spaced apart would you consider your breasts to be?

- ◯ Close together
- ◯ Average
- ◯ Widely spaced (at least 1.5 inches)
- ◯ Unsure

Please refer to image just viewed. Thinking about the shortest distance between where your breasts attach to your chest wall, how widely spaced apart would you consider your breasts to be?

- ◯ Close together
- ◯ Average
- ◯ Widely spaced (at least 3.8 cm)
- ◯ Unsure

Feel free to comment about the width of the space between your breasts.

____

Did you have any surgery to your breast/s before the birth of your first child?

- ◯ Yes
- ◯ No

What type of breast surgery did you have? (Tick all that apply).	☐ Breast augmentation (implant or similar) of both breasts<br>☐ Breast augmentation (implant or similar) of one breast<br>☐ Breast lift<br>☐ Breast reduction<br>☐ Other
What 'other' breast surgery did you have before the birth of your first child?	__________________________________
When did you first have the breast surgery? (Tick all that apply).	☐ Before 8 years of age<br>☐ Between 8 and 19 years of age<br>☐ Between 20 years of age and before being pregnant with my first child<br>☐ When pregnant with my first child<br>☐ Unsure
Please comment about why the breast surgery was done.	__________________________________

Breast shape figure

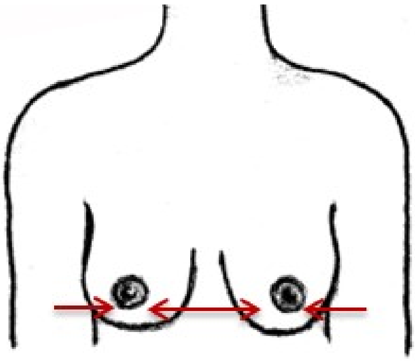

Shape 1 Round breasts with obvious breast tissue in the lower parts

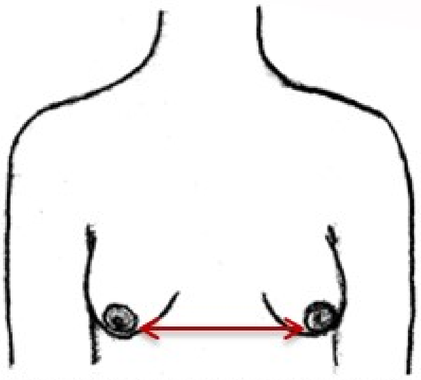

Shape 2 Visually reduced breast tissue in the lower inner part

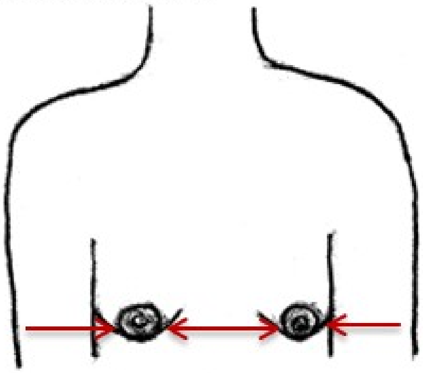

Shape 3 Visually reduced breast tissue in both the lower inner and outer parts

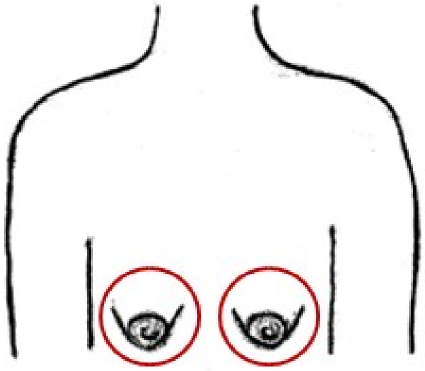

Shape 4 Limited breast tissue overall

Please refer to the figure just viewed. Thinking about how your RIGHT breast looked just before pregnancy with your first child, please indicate which breast shape (1, 2, 3 or 4) your RIGHT breast looked the most like, irrespective of the size of the breast. You can make a guess.	◯ Shape 1<br>◯ Shape 2<br>◯ Shape 3<br>◯ Shape 4<br>◯ None of the above<br>◯ Unsure
Please refer to the figure just viewed. Thinking about how your LEFT breast looked just before pregnancy with your first child, please indicate which breast shape (1, 2, 3 or 4) your LEFT breast looked the most like, irrespective of the size of the breast. You can make a guess.	◯ Shape 1<br>◯ Shape 2<br>◯ Shape 3<br>◯ Shape 4<br>◯ None of the above<br>◯ Unsure
Based on the figure just viewed, please indicate which breast shape (1, 2, 3 or 4) your RIGHT breast looked the most like prior to the breast surgery, irrespective of the size of the breast. You can make a guess.	◯ Shape 1<br>◯ Shape 2<br>◯ Shape 3<br>◯ Shape 4<br>◯ None of the above/Unsure
Based on the figure just viewed, please indicate which breast shape (1, 2, 3 or 4) your LEFT breast looked the most like prior to the breast surgery, irrespective of the size of the breast. You can make a guess.	◯ Shape 1<br>◯ Shape 2<br>◯ Shape 3<br>◯ Shape 4<br>◯ None of the above/Unsure

Now we'd like to ask you some questions about your pregnancy with your first child.

Did you receive any fertility treatment to become pregnant with your first child?

- ◯ No
- ◯ Yes
- ◯ Unsure
- ◯ Prefer not to say

Was the fertility treatment to become pregnant with your first child related (even partly) to female infertility?

- ◯ No
- ◯ Yes
- ◯ Unsure
- ◯ Prefer not to say

What fertility treatment did you receive?

	No	Yes	Unsure	Prefer not to say
Ovulation induction	◯	◯	◯	◯
Artificial insemination	◯	◯	◯	◯
In-vitro fertilisation (IVF)	◯	◯	◯	◯
Intracytoplasmic sperm injection (ICSI)	◯	◯	◯	◯
Donor sperm	◯	◯	◯	◯
Donor eggs	◯	◯	◯	◯
Donor embryos	◯	◯	◯	◯
Unsure	◯	◯	◯	◯
Other	◯	◯	◯	◯

What 'other' fertility treatment did you receive?

____

What was the difference in bra cup size at the end compared to the start of the pregnancy with your first child? (If the answer is different for each breast, answer for the breast that showed the smallest change).

- ◯ No noticeable change in breast size to either breast
- ◯ Increase by less than one cup size
- ◯ Increase by 1 cup size
- ◯ Increase by 2 cup sizes
- ◯ Increase by more than two cup sizes
- ◯ Unsure

Please comment about why you're unsure about any bra cup size change in pregnancy with your first child.

____

Now we'd like to ask you some questions about your medical history.

Have you been medically diagnosed with an underactive thyroid gland (hypothyroidism) before the birth of your first child?
- ◯ No
- ◯ Yes
- ◯ Unsure
- ◯ Prefer not to say

Please comment about why you're unsure if you've been diagnosed with an underactive thyroid gland.
____

When was the underactive thyroid gland diagnosed? (Tick all that apply).
- ☐ Before 8 years of age
- ☐ Between 8 and 19 years of age
- ☐ Between 20 years of age and before being pregnant with my first child
- ☐ When pregnant with my first child
- ☐ Unsure

Have you been medically diagnosed with polycystic ovary syndrome (PCOS) before the birth of your first child?
- ◯ No
- ◯ Yes
- ◯ Unsure
- ◯ Prefer not to say

Please comment why you're unsure if you've been diagnosed with polycystic ovary syndrome.
____

When was the PCOS diagnosed? (Tick all that apply).
- ☐ Before 8 years of age
- ☐ Between 8 and 19 years of age
- ☐ Between 20 years of age and before being pregnant with my first child
- ☐ When pregnant with my first child
- ☐ Unsure

Have you ever been prescribed metformin for PCOS?
- ◯ No
- ◯ Yes
- ◯ Unsure

Do you have type I (juvenile) diabetes which was diagnosed before the birth of your first child?
- ◯ No
- ◯ Yes
- ◯ Unsure
- ◯ Prefer not to say

When was the type I diabetes diagnosed? (Tick all that apply).
- ☐ Before 8 years of age
- ☐ Between 8 and 19 years of age
- ☐ Between 20 years of age and before being pregnant with my first child
- ☐ When pregnant with my first child
- ☐ Unsure

Did you have gestational diabetes (diabetes during pregnancy) with your first child?
- ◯ No
- ◯ Yes
- ◯ Unsure
- ◯ Prefer not to say

Please comment about why you're unsure if you had gestational diabetes when pregnant with your first child.
____

Was the gestational diabetes...	◯ Diet-controlled (without medication)<br>◯ Controlled with medication

For the gestational diabetes, did you ever take any of the following before the birth of your first child?

	Yes	No	Unsure
Metformin	◯	◯	◯
Insulin	◯	◯	◯
Other	◯	◯	◯

What 'other' medication did you take for gestational diabetes when pregnant with your first child?	__________________________________
Have you been diagnosed with type II diabetes before the birth of your first child?	◯ No<br>◯ Yes<br>◯ Unsure<br>◯ Prefer not to say
When was the type II diabetes diagnosed? (Tick all that apply).	☐ Before 8 years of age<br>☐ Between 8 and 19 years of age<br>☐ Between 20 years of age and before being pregnant with my first child<br>☐ When pregnant with my first child<br>☐ Unsure

For the type II diabetes, did you ever take any of the following before the birth of your first child?

	Yes	No	Unsure
Metformin	◯	◯	◯
Insulin	◯	◯	◯
Other	◯	◯	◯

What 'other' medication have you taken for type II diabetes before the birth of your first child?	__________________________________
Did you have high blood pressure during your pregnancy with your first child?	◯ No<br>◯ Yes<br>◯ Unsure<br>◯ Prefer not to say
Were you been prescribed medication for high blood pressure in the pregnancy with your first child?	◯ No<br>◯ Yes<br>◯ Unsure
Before the birth of your first child, had you ever had any problems with your pituitary gland?	◯ No<br>◯ Yes<br>◯ Unsure<br>◯ Prefer not to say
Please comment why you're unsure if you'd had any pituitary gland problems.	__________________________________

Were you diagnosed with any condition as part of an investigation of your low milk supply after discovering you had a low milk supply with your first child?

- ◯ No
- ◯ Yes
- ◯ Unsure
- ◯ Prefer not to say

What condition(s) were you diagnosed with as part of an investigation of your low milk supply after discovering you had a low milk supply with your first child?

	Yes	No	Unsure	Prefer not to say
Thyroid problem	◯	◯	◯	◯
Polycystic ovary syndrome	◯	◯	◯	◯
Type I diabetes	◯	◯	◯	◯
Type II diabetes	◯	◯	◯	◯
Other	◯	◯	◯	◯

What 'other' condition were you diagnosed with?

____

Before the birth of your first child, had you ever had an eating disorder that delayed or stopped you from getting your period?

- ◯ No
- ◯ Yes
- ◯ Unsure
- ◯ Prefer not to say

Please comment about why you're unsure if you have ever had an eating disorder that delayed or stopped you from getting your period.

____

Before the birth of your first child, had you ever been so physically active that it delayed or stopped you from getting your period?

- ◯ No
- ◯ Yes
- ◯ Unsure
- ◯ Prefer not to say

Please comment about why you're unsure if you have ever been so active that it delayed or stopped you from getting your period.

____

Do you have a history of any of the following before the birth of your first child?

	No	Yes	Unsure	Prefer not to say
Congenital syndrome involving your chest? (e.g. Poland, Jeune or ulnar-mammary syndrome, or chest wall deformities such as pectus excavatum or pectus carinatum)	○	○	○	○
Breast hemangioma	○	○	○	○
Congenital adrenal hyperplasia	○	○	○	○
Being treated with radiation to your chest area	○	○	○	○
Any injury (e.g., burn) to your breast/s	○	○	○	○

Please comment about what injury you have had to your breast/s. __________________________________

During your pregnancy with your first child, approximately how many cigarettes did you smoke per day?

- ○ None
- ○ Occasional cigarette
- ○ 1-9
- ○ 10-19
- ○ 20-29
- ○ 30-39
- ○ 40 or more a day
- ○ Stopped when I found out I was pregnant

During your pregnancy with your first child, approximately how many e-cigarettes did you smoke per day?

- ○ None
- ○ Occasional e-cigarette
- ○ 1-9
- ○ 10-19
- ○ 20-29
- ○ 30-39
- ○ 40 or more a day
- ○ Stopped when I found out I was pregnant

Now we'd like to ask you some questions about your family and breastfeeding.

Did your biological mother breastfeed any of her children?	◯ No<br>◯ Yes<br>◯ Unsure
Has your mother reported having difficulty making enough milk?	◯ No<br>◯ Yes<br>◯ Unsure
Do you have at least one biological sister?	◯ No<br>◯ Yes<br>◯ Unsure
Has any biological sister breastfed?	◯ No<br>◯ Yes<br>◯ Unsure
Has any sister reported having difficulty making enough milk?	◯ No<br>◯ Yes<br>◯ Unsure

Now we'd like to ask you some questions about your weight and height.

Please select your height in feet and inches.

◯ 4 feet
◯ 4 feet, 1 inch
◯ 4 feet, 2 inches
◯ 4 feet, 3 inches
◯ 4 feet, 4 inches
◯ 4 feet, 5 inches
◯ 4 feet, 6 inches
◯ 4 feet, 7 inches
◯ 4 feet, 8 inches
◯ 4 feet, 9 inches
◯ 4 feet, 10 inches
◯ 4 feet, 11 inches
◯ 5 feet
◯ 5 feet, 1 inch
◯ 5 feet, 2 inches
◯ 5 feet, 3 inches
◯ 5 feet, 4 inches
◯ 5 feet, 5 inches
◯ 5 feet, 6 inches
◯ 5 feet, 7 inches
◯ 5 feet, 8 inches
◯ 5 feet, 9 inches
◯ 5 feet, 10 inches
◯ 5 feet, 11 inches
◯ 6 feet
◯ 6 feet, 1 inch
◯ 6 feet, 2 inches
◯ 6 feet, 3 inches
◯ 6 feet, 4 inches
◯ 6 feet, 5 inches
◯ 6 feet, 6 inches
◯ 6 feet, 7 inches
◯ 6 feet, 8 inches
◯ 6 feet, 9 inches
◯ 6 feet, 10 inches
◯ 6 feet, 11 inches

What is your height in centimetres? __________________________________

What was your weight in kilograms just before being pregnant with your first child? __________________________________

What was your weight in pounds just before being pregnant with your first child? __________________________________

BMI __________________________________

BMI. In excel, manually calculate BMI by: [weight_pounds/(inches*inches)]*703 __________________________________

Inches calculation. In excel, manually calculate inches from feet and inches question by multiplying feet by 12 and adding inches __________________________________

Which of the following best describes your weight between 8 and 20 years of age?	◯ Underweight<br>◯ Normal weight<br>◯ A little overweight<br>◯ Moderately overweight<br>◯ Very overweight<br>◯ Unsure<br>◯ Prefer not to say<br>◯ Other
Please indicate what you mean by 'other' in relation to your weight between 8 and 20 years of age.	__________________________________

Now we'd like to ask you some questions about any medications you might be taking or may have taken.

Did you ever use of hormonal birth control between 8 and 20 years of age?

- ◯ No
- ◯ Yes
- ◯ Unsure

For what reason(s) did you take hormonal birth control between 8 and 20 years of age?

	No	Yes
Acne	◯	◯
Menstrual cycle problems	◯	◯
To prevent pregnancy	◯	◯
Unsure	◯	◯
Other	◯	◯

What 'other' reason did you have hormonal birth control between 8 and 20 years of age? ______________________________

Have you taken any of the following substances to try to increase your milk supply with your first child?

	No	Yes
Domperidone (Motilium)	○	○
Metoclopramide (Maxolon/Reglan)	○	○
Goat's rue	○	○
Fenugreek	○	○
Blessed thistle	○	○
Brewer's yeast	○	○
Other	○	○

What 'other' substance did you take to try to increase your milk supply with your first child? __________________________________

Now we'd like to ask you some questions about the birthing process with your first child.

Which of the following about labour with your first child is relevant for you?
- ◯ It was spontaneous (that is, it occurred naturally)
- ◯ It was induced (that is, medication was used or had waters broken to start labour)
- ◯ I had no labour (e.g., planned caesarean section)

How was your first baby born?
- ◯ Planned caesarean section
- ◯ Unplanned or emergency caesarean section
- ◯ Assisted vaginal (forceps or vacuum)
- ◯ Vaginal birth

Which of the following pain relief options did you use during labour with your first child?

	No	Yes	Unsure
I had an epidural	◯	◯	◯
I had intravenous (IV) pain or intramuscular (IM) relief (e.g. pethidine or morphine)	◯	◯	◯
I had nitrous oxide gas	◯	◯	◯
Other	◯	◯	◯

What 'other' pain relief did you have during labour with your first child?

____

Did you have a postpartum haemorrhage (blood loss >500mL) during the birthing process with your first child?
- ◯ No
- ◯ Yes
- ◯ Unsure

Did you have a postpartum haemorrhage (blood loss >2 cups) during the birthing process with your first child?
- ◯ No
- ◯ Yes
- ◯ Unsure

Do you know what volume of blood was lost?
- ◯ No
- ◯ Yes

What volume of blood (in mL) was lost after the birth of your first child?

____

What volume of blood (in mL) was lost after the birth of your first child?

____

What volume of blood (in ounces) was lost after the birth of your first child?

____

Did you need a blood transfusion or iron infusion after the birth of your first child?
- ◯ No
- ◯ Yes

Did you need a blood transfusion or iron infusion after the birth of your first child?
- ◯ No
- ◯ Yes

Was all of your placenta removed during the birth of your first child?	◯ No. It was discovered that not all of my placenta was removed during the birth of my first child<br>◯ Yes. All of my placenta was removed during the birth of my first child<br>◯ Unsure. I'm unsure if all of my placenta was removed during the birth of my first child
Please comment why you're unsure if all of your placenta was removed during the birth of your first child.	______________________________

Now we'd like to ask you some questions about feeding your first child.

Was your first child born with a condition which made it more difficult for them to remove milk while breastfeeding? (e.g., a cleft palate or heart condition).

◯ Yes
◯ No

What condition was your first child born with that made it more difficult for them to remove milk while breastfeeding?

____

Thinking back to when you were pregnant with your first child, were you planning to breastfeed your first baby at all (even if only for a few days, or in combination with formula)?

◯ No
◯ Yes

How long were you planning to breastfeed your first baby for? (Give estimate if unsure).

____

How long were you planning to exclusively breastfeed your first baby for (‘exclusive’ breastfeeding means that your baby is fed only breastmilk)? (Give estimate if unsure).

____

When was colostrum first removed (either with expressing or breastfeeding) from your breasts after your first child was born? (Colostrum is the first milk a mother's breasts make in the early days after their baby is born).

◯ < 1 hour
◯ 1 hour to 2 hours
◯ > 2 hours to 4 hours
◯ > 4 hours to 6 hours
◯ > 6 hours
◯ Unsure
◯ It was never removed

How was colostrum removed for the first time after your first child was born?

◯ Breastfeed
◯ Hand expressing
◯ Breastfeed and hand expressing
◯ Breast pump
◯ Unsure

Did your first child first receive any infant formula or donor milk before your milk came in? (Milk 'coming in' refers to the time when a mother's breasts become noticeably fuller after the birth of their baby).

◯ No
◯ Yes
◯ Unsure

What was the main reason for your first child receiving formula or donor milk before your milk came in?

____

Now we'd like to ask you some questions about breast changes after the birth of your first child.

We are interested in the breast changes you noticed after the birth of your first child.

If you think of the breast changes you felt on a scale of 1 to 5, where 1 is no change, 3 is noticeably fuller, and 5 is uncomfortably full. When did you first feel that your breasts were 'noticeably fuller' (i.e., a "3" on the scale) after the birth of your first child?

- ◯ Within the first week
- ◯ After the first week
- ◯ Breasts never became noticeably fuller
- ◯ Unsure

Please comment about your breasts never becoming noticeably fuller.

____

Please comment about why you're unsure about your breasts becoming noticeably fuller.

____

Did your breasts feel noticeably fuller within the first 72 hours after your first child was born?

- ◯ No
- ◯ Yes
- ◯ Unsure

Thank you for completing these questions. Finally, we’d like to give you the chance to share your experience with us.

Please explain, in your own words, your personal story of how your low milk supply was discovered or diagnosed with your first child. ____
