# Supplementary material for: Breast hypoplasia markers among women who report insufficient milk production: A retrospective online survey
Source: PLoS One. 2024 Feb 29;19(2):e0299642. doi: 10.1371/journal.pone.0299642 (PMC10903845; doi:10.1371/journal.pone.0299642)
Supplement: S2 File — (PDF) [file pone.0299642.s011.pdf]

Breast Variations Among Women With Low Milk Supply - Eligibility check

Thank you for your interest in this study. Please complete the short screening check below to see if you are eligible to take part.

The aim of this study is to find out how common certain breast features are among women who report low milk supply and to find out if certain breast features are linked with other health conditions.

Is your usual country of residence the United States of America, Australia or the United Kingdom?	◯ Yes<br>◯ No
Are you at least 18 years of age?	◯ Yes<br>◯ No
How long ago did you give birth to your first child? (Please answer for live births only).	◯ Between 0 and 5 years ago<br>◯ More than 5 years ago
Are you experiencing low milk supply with your first child? Or, did you have low milk supply with your first child?	◯ Yes<br>◯ No<br>◯ Unsure
Before you recognised you had a low milk supply with your first child, did you attempt to breastfeed or express breastmilk at least 6 times each day (i.e., 6 times over 24 hours) from each breast on most days?	◯ Yes<br>◯ No
Was your first child born before 37 weeks (that is, preterm)?	◯ Yes<br>◯ No
Was your first child born as a singleton birth? (i.e., your first child was not a part of a multiple birth such as a twin or triplet etc).	◯ Yes<br>◯ No
Were you separated from your first child for more than 24 hours during your maternity hospital stay? (e.g., you or your child required specialised hospital care in the first few days after giving birth?)	◯ Yes<br>◯ No
