# Supplementary material for: Breast hypoplasia markers among women who report insufficient milk production: A retrospective online survey
Source: PLoS One. 2024 Feb 29;19(2):e0299642. doi: 10.1371/journal.pone.0299642 (PMC10903845; doi:10.1371/journal.pone.0299642)
Supplement: Attachment — Submitted filename: Kuznetsov 2017- kliniko-patogenetichni-aspekti.pdf [file pone.0299642.s014.pdf]

УДК 618.2-055.25
Кузнєцов В.Г.

КЛІНІКО-ПАТОГЕНЕТИЧНІ АСПЕКТИ ГІПОГАЛАКТІЇ У ПОРОДІЛЬ

Перший Київський медичний коледж

В аналітичному огляді клінічних досліджень розкривається проблема розладів лактаційної функції у породіль. Описані основні патофізіологічні чинники гіпогалактії, механізми безпосереднього та опосередкованого впливу на рівень лактації, їх синергії, методи профілактики і корекції порушеної лактаційної функції.

Ключові слова: молочна залоза, гіпогалактія.

Недостатність лактаційної функції молочної залози є одним з найбільш частих порушень у породіль, які мають місце майже у третини жінок. В клініці первинна гіпогалактія зустрічається не більше ніж у 3-8% жінок, а всі інші випадки є наслідком гормональної дисфункції, що виникли у жінки під час вагітності і мають низку патогенетичних чинників [1].

Згідно МКБ-10 порушення лактаційної функції класифікують на первинну агалактію (О92.2), гіпогалактію (О92.3), факультативну, вторинну агалактію (О92.5) і галакторею (О92.6) [2]. Залежно від дефіциту молока щодо добової потреби дитини виділяють 4 ступеня гіпогалактії:

I – дефіцит молока не більше 25%;

II – дефіцит молока на 26-50%;

III – дефіцит молока на 51-75%;

IV – дефіцит молока більш ніж на 75% [3].

За статистичними даними страждають від недостатності лактації близько 25-80% серед породіль [4,5]. В структурі захворюваності гіпогалактія I ступеня зустрічається у 33,6% породіль, II ступеня – у 16,4% жінок, III ступеня і агалактія – у 37,3% [6]. При цьому в перші 2-4 діб після пологів у молодих жінок, які вперше народжують гіпогалактію слід відрізняти від тимчасової недостатньої секреції молока, оскільки при правильному режимі і догляді за молочними залозами лактація швидко відновлюється до фізіологічної норми [7]. Іншою проблемою є так звана «лактаційна криза», яка виникає із ростом дитини і зумовлена невідповідністю рівня лактації із набором ваги дитини. Як і у випадку післяродової гіпогалактії «лактаційні кризи» пов’язана з гормональними змінами і спостерігаються, як правило, в перші 3 місяці лактації. У всіх інших випадках недостатність лактаційної функції обумовлена патологією з боку соматичного і репродуктивного здоров'я жінок, стресових чинників, високим рівнем тривожності і невротичних проявів [8].

Частою причиною гіпогалактії є пізній початок (через 3-12 годин і більше після пологів) годування новонародженого [9], екстрагенітальна патологія і обтяжений акушерсько-гінекологічний анамнез і ускладненнями, що виникли під час вагітності, пологів і у післяпологовому періоді, а також зі зниженням рівня фізичного і психічного здоров'я матері [10]. Іншою причиною є гестози, що підвищують вірогідність гіпогалактії на 23,7% [11].

До групи з високим ризиком гіпогалактії входять жінки зі слабкістю родової діяльності, які отримували стимуляцію під час пологів (вітамінно-гормональну терапію, окситоцин, естрогени, простагландини) [12]. Після фармакологічної стимуляції пологів в перші дні після пологів відмічають дефіцит лактації [13]. Гіпогалактія також може виникнути у відповідь на стресові обриви дитячо-материнського емоційного зв'язку, в основі яких порушення окситоцинового рефлексу лактації.

Питома вага жінок із нормальною лактацією статистично значимо вище серед породіль після пологів без ускладнень в порівнянні з жінками, у яких пологи ускладнилися аномалією родової діяльності, гестозом різного ступеня тяжкості, гіпоксією плода, кровотечами в послідовно і ранньому післяпологовому періоді, оперативними втручаннями [13]. Все це вказує на те, що молочні залози дуже чутливі до впливу зовнішніх і внутрішніх чинників, а стан соматичного і репродуктивного здоров'я жінок має практичне значення для прогнозування лактації.

За даними клінічних спостережень, 68,2% жінок з гіпогалактією мали в анамнезі соматичні захворювання (хронічний тонзиліт, патологію серцево-судинної системи, нирок, анемію, ювенільні маткові кровотечі, алергічні захворювання, ендокринну патологію) [15]. У 26,6% жінок реєструють захворювання серцево-судинної системи [16], а частота виникнення гестозу у жінок з артеріальною гіпертензією в анамнезі складає від 5% до 89% і відрізняється між дослідженнями різних авторів. Виділено чотири основні гіпертензивні розлади під час вагітності: хронічна гіпертонія, гестаційна гіпертензія, прееклампсія та хронічна гіпертонія з прееклампсією [17]. Встановлено кореляцію гіпотонії з гестозами, прееклампсією і еклампсією, прогресуючою плацентарною недостатністю [18]. У жінок з гіпотонією частота гіпогалактії становить 63-75%. Відзначається погіршення якісного складу молока, що проявляються у вигляді зниження загальної енергетичної ємності молока, його калорійності за рахунок зменшення вмісту основних нутрієнтів (білків, жирів і вуглеводів) [15]. В загальній структурі гіпогалактії у жінок з судинними розладами прееклампсія є одним з найбільш частих розладів, що впливає на вагітність, зі значним ризиком для здоров’я матері і плоду, при цьому абсолютно не встановленим залишилася їх роль у виникненні порушень лактаційної функції [19].

У жінок з залізодефіцитною анемією (ЗДА) та

патологіє щитовидної залози також виявлено порушення лактаційної функції: нормальна кількість секрету молочних залоз відзначено тільки у 11,3% породіль, в той час як при гіпотиреозі – у 23,4%, при ЗДА – у 30%, у здорових породіль – 78%. У породіль з гіпотиреозом в поєднанні з ЗДА відзначені висока частота гіпогалактії II і III ступенів [8].

Гіпогалактія може бути обумовлена морфологічною або функціональною неповноцінністю молочних залоз. Найбільшого розвитку молочні залози досягають на момент пологів, а секреція молока визначаються складним механізмом нейрогормональної регуляції (пролактин, окситоцин, вазопресин). Повний цикл лактації включає ряд взаємозв'язаних процесів: мамогенез – розвиток молочної залози, лактогенез – ініціація секреції молока після пологів, лактопоез – розвиток і підтримка секреції молока [20]. Ріст, розвиток молочної залози і лактація перебуває під контролем нейрогормонів, а саме пролактину і окситоцину. Останній, через здатність стимулювати міометрій, почали використовувати в якості медикаментозного методу стимуляції пологів. Проте, як показав багаторічний досвід, відмічається різна чутливість організму жінок під час пологів до окситоцину, що безпосередньо визначається фізіологією появи окситоцинових рецепторів. На початку пологів концентрація окситоцинових рецепторів буває істотно вище, ніж у жінок без родової діяльності. В ранні терміни вагітності виявлено високу концентрацію окситоцину в плазмі крові, яка не приводить до підвищення активності міометрія. Це пояснюється низьким рівнем окситоцинових рецепторів в міометрії в ці терміни. Суттєве підвищення їх кількості в міометрії може призводити до активації матки без зміни рівня окситоцину в плазмі крові. На час розкриття шийки матки на 7 см і більше, а також і при відсутності ефекту від медикаментозної стимуляції пологів, виявлено низьку концентрацію окситоцинових рецепторів. Концентрація окситоцинових рецепторів залишається низькою протягом всієї вагітності і раптово підвищується за кілька годин до пологів і тримається на максимальному рівні під час пологів і через 1-2 дні після пологів знижується до передпологового рівня. У експериментальних дослідженнях, що проводились на гіпертензивних щурів, встановлено зменшений рівень експресії вазопресину і окситоцину і відповідно менший рівень лактації у щурів після народження. Біохімічний склад молока також виявився збіднений порівняно із нормотензивними тваринами [21]. При цьому екзогенне введення окситоцину дозволяє зменшити рівень артеріального тиску, проте вплив гормону на лактаційну функцію за таких умов залишається невстановленим.

Нажаль, існуючі медикаментозні засоби профілактики та корекції гіпогалактії мають ряд побічних реакцій і протипоказань до застосування, що істотно знижує їх можливість застосування у породіль [22]. Більшість немедикаментозних методів корекції лактаційної функції застосовуються після встановлення діагнозу гіпогалактії, коли дефіцит молока і недостатність лактаційної функції стають гострою проблемою у вигодовуванні дитини [23]. Тобто профілактичні і терапевтичні заходи щодо покращення лактації недостатньо ефективні, і головне - патогенетичні причини гіпогалактії в кожному випадку як правило залишаються невстановленими і не до кінця вивченими.

Єдиним напрямком поліпшення лактації у породіль є проведення заходів, спрямованих на раціоналізацію харчування. Дієтотерапія є головним напрямком у підтриманні здоров'я породіль та профілактики недостатності лактації, корекції біохімічного складу молока. Раціональна профілактика спрямована на проведення курсів метаболічної терапії (антиоксиданти, цитопротектори, антагоністи дофаміну, адаптогени, імуномодулятори), профілактику і лікування залізодефіцитної анемії, фетоплацентарної недостатності [24].

Таким чином, узагальнюючи вищеописані клінічні спостереження, слід відмітити, що поява недостатності лактаційної функції у породіль пов'язана з гормональними і соматичними чинниками, синергією цих чинників. Дослідження патогенетичних чинників гіпогалактії, розробки ефективних методів профілактики і корекції порушеної лактаційної функції у породіль з супутніми патологічними станами мають важливе практичне значення.

Література

1. Лук'яненко М. В. Прогнозування та профілактика гіпогалактії у жінок з пізнім прикладанням новонародженого до грудей : автореф. дис. на здобуття наук ступеня канд. мед. наук : спец. 14.01.01 «Акушерство та гінекологія» / М. В. Лук'яненко. – Київ, 2004. – 17 с.
2. Шишак О.І. Ідентифікаційні розлади в діаді як чинник етіопатогенезу вторинної гіпогалактії / О.І. Шишак // Український науково-медичний молодіжний журнал. – 2011. – Вип.3. – С. 63-66.
3. Борис О.М. Современный подход к лечению вторичной гипогалактии в амбулаторной практике / О.М. Борис, Л.М. Онищик, Г.В, Сотниченко, И. А. Гак // Науковий журнал МОЗ України. – 2014. - № 1 (5). – С. 93-99.
4. Литвинова Е.В. Оптимизирующее влияние лазеротерапии на раневой процесс и реактивность организма родильниц с гестозом / Е.В. Литвинова / Архив клинической и экспериментальной медицины. — 2003. — Т. 12, № 1. - С. 57-61.
5. Яхъева М.Р. Особенности лактации у родильниц Чеченской Республики и пути ее оптимизации : диссертация ... кандидата медицинских наук : 14.01.01 «Акушерство та гінекологія» / Яхъева М.Р. - Москва, 2010. - 165 с.
6. Есартия М.А. Прогнозирование, профилактика и лечение ранних нарушений лактации : автореферат дис. ... кандидата медицинских наук : 14.00.01 «Акушерство та гінекологія» / М.А. Есартия.- Самара, 2007.- 28 с.:
7. Яковлева Л.В. Оценка физического развития детей первого года жизни в зависимости от вида вскармливания / Л.В. Яковлева, А.А. Латыпова, В.Р. Башаров, Л.Р. Нургалиева / Современные проблемы науки и образования. – 2014. – № 3. Електронний ресурс: http://www.science-education.ru/ru/article/view?id=13522.
8. Омарова М.Ш. Лактационная функция у женщин при сочетании гипотиреоза и железодефицитной анемии : диссертация ... кандидата медицинских наук : 14.00.01 «Акушерство та гінекологія» / Омарова М.Ш. - Ростов-на-Дону, 2008. - 180 с.
9. Трусова О.Ю. Причины и факторы риска перевода детей на искусственное вскармливание: программа поддержки естественного вскармливания : диссертация ... кандидата медицинских наук : 14.00.09 «Педіатрія» / Трусова О.Ю. – 2007. - 261 с.

10. Sergienko S.N. The pathogenetic role of the immune and microcirculatory disorders in chronic diseases of the hepatobiliary system in women of child-bearing age living in the industrial region of the Donets Basin / S.N. Sergienko // Lik Sprava. – 1997. – 6. – P. 45-48.
11. Марков А. Г. Содержание простагландина Р2-альфа и пролактина в плазме крови и молоке у женщин в период лактогенеза / А. Г. Марков, Е. Н. Парийская, М.А. Кучеренко // Акушерство и гинекология. — 2006. — № 2. — С.33-35.
12. Odent M.R. Synthetic oxytocin and breastfeeding: reasons for testing and hypothesis / M.R. Odent / Med Hypotheses. – 2013. – Vol. 81(5). – P. 889-891.
13. Герасимович Г. И. Функция лактации и грудное вскармливание / Г. И. Герасимович // Здравоохранение. — 2003. — № 11. — С. 26–33.
14. Нелюбова А.Б. Гипогалактия. Диагностика, профилактика и лечение : автореферат дис. ... канд. мед. наук : 14.01.01 «Акушерство та гінекологія» / А. Б. Нелюбова. - Омск, 2010. - 22 с.
15. Гарунов Э.Г. Влияние артериальной гипотонии на лактационную функцию : диссертация ... кандидата медицинских наук : 14.00.01 «Акушерство та гінекологія» / Гарунов Э.Г. - Волгоград, 2006.
16. Бакун О.В. Гіпогалактія як прояв ускладнень післяпологового періоду / О.В. Бакун, В.Г. Купчанко, А.М. Бербець, О.А. Андрієць // Електронний ресурс: http://dspace.bsmu.edu.ua:8080/xmlui/handle/123456789/1649.
17. Countouris M.E. Effects of lactation on postpartum blood pressure among women with gestational hypertension and preeclampsia / M.E. Countouris, E.B. Schwarz, B.C. Rossiter [et al.] // Am. J. Obstet. Gynecol. – 2016. - pii: S0002-9378(16)00388-4.
18. Гутикова Л. В. Гормональная регуляция лактации у родильниц, перенесших гестоз / Л. В. Гутикова // Журнал Гродненского государственного медицинского университета: ежеквартальный научно-практический журнал. - 2010. - №1. - С. 68-69
19. Marik P.E. Hypertensive disorders of pregnancy / P.E. Marik // Postgrad Med. – 2009. – Vol. 121(2). – P. 69-76.
20. Ольшевский В. С. Нейроэндокринная регуляция лактационной функции / В.С. Ольшевский / Питання експериментальної та клінічної медицини : зб. наук. статей - 2012. - Вип. 16, т. 2. - С .176-183.
21. Wlodek M.E. Impaired mammary function and parathyroid hormone-related protein during lactation in growth-restricted spontaneously hypertensive rats / M.E. Wlodek, K.T. Westcott, A. Serruto [et al.] // J Endocrinol. - 2003. – Vol. 178(2). – P. 233-245.
22. Mangesi L. Treatments for breast engorgement during lactation / L. Mangesi, I. Zakarija-Grkovic // Cochrane Database Syst Rev. – 2016. – Vol. 28;(6). - CD006946.
23. Амирханова М.И. Нарушение лактационной функции у многорожавших женщин с гестозом : диссертация ... кандидата медицинских наук : 14.00.01 «Акушерство и гинекология» / М.И. Амирханова - Ростов-на-Дону, 2002. - 147 с.
24. Абдуллаева М.З. Особенности гестации и лактации у юных первородящих : автореферат дис. ... кандидата медицинских наук : 14.00.01 «Акушерство и гинекология» / М.З. Абдуллаева. - Ростов-на-Дону, 2007. - 25

Реферат

КЛИНИКО-ПАТОГЕНЕТИЧЕСКИЕ АСПЕКТЫ ГИПОГАЛАКТИИ У РОЖЕНИЦ

Кузнецов В.Г.

Ключевые слова: молочная железа, гипогалактия.

В аналитическом обзоре научных клинических исследований раскрывается проблема расстройств лактационной функции у рожениц. Описаны основные патофизиологические факторы гипогалактии, механизмы непосредственного и косвенного влияния на уровень лактации, их синергии, методы профилактики и коррекции нарушенной лактационной функции.

Summary

CLINICAL AND PATHOGENETIC ASPECTS OF HYPOGALACTIA IN POST-PARTURIENT WOMEN

Kuznetsov V.G.

Key words: breast, mammary glands, hypogalactia.

This article presents the analytical review of clinical studies related to the problems of galactosis in post-parturient women. The basic pathophysiological factors of hypogalactia, mechanisms of direct and indirect effects on the level of lactation, their synergy, and methods of prevention and correction of lactation dysfunction were described.
