# Supplementary material for: Breast hypoplasia markers among women who report insufficient milk production: A retrospective online survey
Source: PLoS One. 2024 Feb 29;19(2):e0299642. doi: 10.1371/journal.pone.0299642 (PMC10903845; doi:10.1371/journal.pone.0299642)
Supplement: Attachment — Submitted filename: Kuznetsov 2017- Clinical and pathogenetic asp.docx [file pone.0299642.s015.docx]

**CLINICAL-PATHOGENETIC ASPECTS OF HYPOGALACTIA IN THE POSTPARTUM WOMEN**

Kuznetsov VG	The First Kyiv Medical College // Translated by Vika Nesterova 3-29-19

In this analytical review we describe main pathophysiological factors of hypogalactia, their direct and indirect influences on lactation, synergy of these factors, and methods of prevention and correction of impaired lactation function.

Impairments of lactation function of the breast is one of the most frequent problems in the postpartum period, occurring in almost a third of all women. Clinically, primary hypogalactia occurs in no more than 3-8% of women, all other cases are results of hormonal dysfunctions experienced by women during their pregnancies and there are a number of pathogenetic factors [1].

According to ICD-10, impairments of lactation function are classified as primary agalactia (O92.2), hypogalactia (O92.3), facultative, secondary agalactia (O92.5), and galactorrhea (O92.6) [2]. There are 4 degrees of hypogalactia depending on the shortage of milk for the child’s daily needs:

I - milk deficit is less than 25% of daily volume;

II - milk deficit is 26-50%;

III - milk deficit is 51-75%;

IV - milk deficit is greater than 75% [3].

According to statistical data, about 25-80% of lactating women suffer from lactation insufficiency/low milk supply [4,5]. In the structure of morbidity, I^st^ degree hypogalactia occurs in 33.6% of the mothers, II degree - in 16.4% of women, III degree and agalactia - in 37.3% [6]. At the same time, hypogalactia should be differentiated from the temporary inadequate secretion of milk in young primiparae in the first 2-4 days after childbirth, because in their case lactation rapidly improves to the physiological norm with proper regimen and care of the mammary glands [7]. Another problem is the so-called "lactation crisis" [growth spurts – VN], which arises from the growth of the child and is caused by [temporary] inconsistency of the maternal milk supply with the rapid weight gain of the child. As in the case of postpartum hypogalactia, "lactation crises" are associated with hormonal changes and they usually happen in the first 3 months of lactation. In all other cases, the lack of lactational function is due to pathologies of the somatic and reproductive health of women, stress factors, high levels of anxiety and neurotic manifestations [8]. The frequent cause of hypogalactia is the late start of breastfeeding (after 3-12 hours or more after delivery [9], extragenital pathology and maternal obstetric and gynecological health problems, complications that arose during pregnancy, childbirth and in the postpartum period, as well as with the deterioration of maternal physical and mental health [10]. Another cause is gestosis, which increases the chances of hypogalactia by 23.7% [11].

The high-risk group for hypogalactia includes women with a slow labor progress, who received augmentation during labor (vitaminal-hormonal therapy, oxytocin, estrogens and prostaglandins) [12]. Pharmacological labor augmentation is associated with the deficiency of lactation function in the first days after birth [13]. Hypogalactia can also occur in response to the stressful interruptions of child-maternal emotional connection (probably because of disruption of oxytocin reflex).

The proportion of women with normal lactation is significantly higher among women with normal childbirth experience without complications, compared to women whose delivery was complicated by abnormal labor, gestosis of varying degrees of severity, fetal hypoxia, postpartum hemorrhages, and surgical interventions [13]. All this indicates that the mammary glands are very sensitive to the external and internal influences, and the state of somatic and reproductive health of women is of practical importance for predicting lactation [success].

According to clinical observations, 68.2% of women with hypogalactia had a history of somatic diseases (chronic tonsillitis, pathology of the cardiovascular system, of kidneys, anemia, juvenile uterine bleeding, allergic diseases, and endocrine pathology) [15]. 26,6% of women report diseases of the cardiovascular system [16].

In women who have arterial hypertension in anamnesis incidence of gestosis ranges from 5% to 89% (data differ between different authors). There are four main hypertension disorders during pregnancy: chronic hypertension, gestational hypertension, preeclampsia and chronic hypertension with preeclampsia [17]. Correlation of hypotension with gestosis, preeclampsia and eclampsia, progressive placental insufficiency [18] was established. Women with hypotension had hypogalactia in 63-75% of cases. Deterioration of milk quality was observed: decreased total energy and decreased caloric content because of reduced proteins, fats and carbohydrates [15].

In women with vascular disorders who have low milk supply, preeclampsia is one of the most common disorders affecting pregnancy, with a significant risk to the health of the mother and the fetus. Its influence on lactation function remains uncertain [19]

In women who had both iron deficiency anemia (IDA) and pathology of the thyroid gland, milk supply was also impaired: normal supply was noted only in 11.3% of the mothers, whereas 23.4% of women with hypothyroidism only had normal supply, 30 % of women with IDA only had normal supply, healthy controls had normal supply in 78%. Women who had both hypothyroidism and IDA during their pregnancy very frequently had II and III degree hypogalactia [8].

Hypogalactia also may occur because of morphological or functional impairments of the mammary glands. Mammary glands reach their full development close to the delivery time, and milk secretion is regulated by complex neurohormonal mechanisms (prolactin, oxytocin and vasopressin). Full cycle of lactation involves several interconnected processes: mammogenesis - development of the mammary gland, lactogenesis - initiation of secretion of milk after delivery, lactopoesis - development and maintenance of milk supply [20]. Growth and development of the mammary gland and lactation are under control of neurohormones, namely prolactin and oxytocin. Oxytocin is used for labor induction and augmentation due to its ability to stimulate myometrium. However, as shown by many years of experience, women’s sensitivity to oxytocin during labor differs. The cause of these differences is the changes in quantity of oxytocin receptors. At the beginning of labor women have significantly more oxytocin receptors than women not in labor. In the early stages of pregnancy high concentrations of oxytocin in blood plasma do not lead to increased myometrium activity. This is because at early stages of pregnancy myometrium has low number of oxytocin receptors. Significant increase in their number in myometrium can lead to uterus activation even when plasma oxytocin levels remain constant. Low quantity of oxytocin receptors was detected at the time of the cervical dilation 7 cm or more, as well as in situation when there is no response to labor augmentation. Concentration of oxytocin receptors remains low throughout pregnancy and suddenly increases several hours before childbirth and remains at the maximum level during labor and after 1-2 days post delivery decreases to pre-pregnancy levels.

In experimental studies on hypertensive rats, reduced levels of expression of vasopressin and oxytocin were detected as well as lower milk supply after birth. Biochemical composition of milk was also impaired in comparison with normotensive animals [21]. In these animals, use of exogenous oxytocin can reduce blood pressure, but whether it can also influence lactation remains unidentified.

Unfortunately, existing pharmacological methods for prophylaxis and correction of hypogalactia have a number of side effects and contraindications. This significantly reduces their potential for use in postpartum women [22]. Most non-pharmacological methods for increasing milk supply are applied only after established diagnosis of hypogalactia, when low supply and deficiency of lactation function already constitute a problem[23]. The problem is that preventive and therapeutic measures to improve lactation are not effective enough, and most importantly, pathogenetic causes of hypogalactia in each case usually remain unidentified and not fully studied.

The only way to improve lactation in postpartum women is measures aimed at improving maternal nutrition. Diet therapy is the main method for maintaining maternal health, prevention of lactation insufficiency and for correction of the biochemical composition of milk. Rational prophylaxis includes courses of metabolic therapy (antioxidants, cytoprotectors, dopamine antagonists, adaptogens, immunomodulators), prevention and treatment of iron deficiency anemia and fetoplacental insufficiency [24].

In summary it should be noted that insufficiency of lactation function in postpartum period is associated with combination and synergy of hormonal and somatic factors. Studies of pathogenetic factors of hypogalactia, development of effective methods of prophylaxis and correction of impaired lactation function in women with associated pathological conditions are very important.

Literature

1. Лук’яненко М. В. Прогнозування та профілактика гіпогалактії у жінок з пізнім прикладанням новонародженого до грудей : авто-реф. дис. на здобуття наук ступеня канд. мед. наук : спец. 14.01.01 «Акушерство та гінекологія» / М. В. Лук’яненко. – Київ, 2004. – 17 с.

2. Шишак О.І. Ідентифікаційні розлади в діаді як чинник етіопатогенезу вторинної гіпогалактії / О.І. Шишак // Український науково-медичний молодіжний журнал. – 2011. – Вип.3. – С. 63-66.

3. Борис О.М. Современный подход к лечению вторичной гипогалактии в амбулаторной практике / О.М. Борис, Л.М. Онищик, Г.В, Сотниченко, И. А. Гак // Науковий журнал МОЗ України. – 2014. - № 1 (5). – С. 93-99.

4. Литвинова Е.В. Оптимизирующее влияние лазеротерапии на раневой процесс и реактивность организма родильниц с гестозом / Е.В. Литвинова / Архив клинической и экспериментальной медицины. — 2003. — Т. 12, № 1. - С. 57-61.

5. Яхъева М.Р. Особенности лактации у родильниц Чеченской Республики и пути ее оптимизации : диссертация ... кандидата медицинских наук : 14.01.01 «Акушерство та гінекологія» / Яхъева М.Р. - Москва, 2010. - 165 с.

6. Есартия М.А. Прогнозирование, профилактика и лечение ранних нарушений лактации : автореферат дис. ... кандидата ме-дицинских наук : 14.00.01 «Акушерство та гінекологія» / М.А. Есартия.- Самара, 2007.- 28 с.:

7. Яковлева Л.В. Oценка физического развития детей первого го-да жизни в зависимости от вида вскармливания / Л.В. Яковлева, А.А. Латыпова, В.Р. Башаров, Л.Р. Нургалиева / Современные проблемы науки и образования. – 2014. – № 3. Електрон-ний ресурс: http://www.science-education.ru/ru/article/view?id=13522.

8. Омарова М.Ш. Лактационная функция у женщин при сочетании гипотиреоза и железодефицитной анемии : диссертация ... кандидата медицинских наук : 14.00.01 «Акушерство та гінекологія» / Омарова М.Ш. - Ростов-на-Дону, 2008. - 180 с.

9. Трусова О.Ю. Причины и факторы риска перевода детей на искусственное вскармливание: программа поддержки естественного вскармливания : диссертация ... кандидата медицинских наук : 14.00.09 «Педіатрія» / Трусова О.Ю. – 2007. - 261 с

Sergienko S.N. The pathogenetic role of the immune and microcir-culatory disorders in chronic diseases of the hepatobiliary system in women of child-bearing age living in the industrial region of the Donets Basin / S.N. Sergienko // Lik Sprava. – 1997. – 6. – P. 45-48.

11. Марков А. Г. Содержание простагландина Р2-альфа и пролак-тина в плазме крови и молоке у женщин в период лактогенеза / А. Г. Марков, Е. Н. Парийская, М.А. Кучеренко // Акушерство и гинекология. — 2006. — № 2. — С.33-35.

12. Odent M.R. Synthetic oxytocin and breastfeeding: reasons for testing and hypothesis / M.R. Odent / Med Hypotheses. – 2013. – Vol. 81(5). – P. 889-891.

13. Герасимович Г. И. Функция лактации и грудное вскармливание / Г. И. Герасимович //Здравоохранение. — 2003. — № 11. — С. 26–33.

14. Нелюбова А.Б. Гипогалактия. Диагностика, профилактика и лечение : автореферат дис. ... канд. мед. наук : 14.01.01 «Акушерство та гінекологія» / А. Б. Нелюбова. - Омск, 2010. - 22 с.

15. Гарунов Э.Г. Влияние артериальной гипотонии на лактационную функцию : диссертация ... кандидата медицинских наук : 14.00.01 «Акушерство та гінекологія» / Гарунов Э.Г. - Волгоград, 2006.

16. Бакун О.В. Гіпогалактія як прояв ускладнень післяпологового періоду / О.В. Бакун, В.Г. |Kупчанко, А.М. Бербець, О.А. Андрі-єць // Електронний ресурс: http://dspace.bsmu.edu.ua:8080/xmlui/handle/123456789/1649.

17. Countouris M.E. Effects of lactation on postpartum blood pressure among women with gestational hypertension and preeclampsia.E. Countouris, E.B. Schwarz, B.C. Rossiter [et al.] // Am. J. Obstet. Gynecol. – 2016. - pii: S0002-9378(16)00388-4.

18. Гутикова Л. В. Гормональная регуляция лактации у родильниц, перенесших гестоз / Л. В. Гутикова // Журнал Гродненского го-сударственного медицинского университета: ежеквартальный научно-практический журнал. - 2010. - №1. - С. 68-69

19. Marik P.E. Hypertensive disorders of pregnancy / P.E. Marik // Postgrad Med. – 2009. – Vol. 121(2). – P. 69-76.

20. Ольшевский В. С. Нейроэндокринная регуляция лактационной функции / В.С. Ольшевский / Питання експериментальної та клінічної медицини : зб. наук. статей - 2012. - Вип. 16, т. 2. - С .176-183.

21. Wlodek M.E. Impaired mammary function and parathyroid hor-mone-related protein during lactation in growth-restricted sponta-neously hypertensive rats / M.E. Wlodek, K.T. Westcott, A. Serruto [et al.] // J Endocrinol. - 2003. – Vol. 178(2). – P. 233-245.

22. Mangesi L. Treatments for breast engorgement during lactation / L. Mangesi, I. Zakarija-Grkovic // Cochrane Database Syst Rev. – 2016. – Vol. 28;(6). - CD006946.

23. Амирханова М.И. Нарушение лактационной функции у много-рожавших женщин с гестозом : диссертация ... кандидата ме-дицинских наук : 14.00.01 «Акушерство и гинекология» / М.И. Амирханова - Ростов-на-Дону, 2002. - 147 с.

24. Абдуллаева М.З. Особенности гестации и лактации у юных пе-рвородящих : автореферат дис. ... кандидата медицинских наук : 14.00.01 «Акушерство и гинекология» / М.З. Абдуллаева. - Ростов-на-Дону, 2007. – 2

Google Translate:

1. Lukyanenko MV Prediction and prevention of hypogalactia in women with late application of the newborn to the breast: auto-ref. dis. for the degree of Cand. honey. Science: special. 14.01.01 "Obstetrics and gynecology" / MV Lukyanenko. - Kyiv, 2004. - 17 p.

2. Shishak OI Identification disorders in the dyad as a factor in the etiopathogenesis of secondary hypogalactia / O.I. Shyshak // Ukrainian scientific and medical youth journal. - 2011. - Issue 3. - P. 63-66.

3. Boris OM Modern approach to the treatment of secondary hypogalactia in outpatient practice / OM Boris, L.M. Onyshchyk, GV, Sotnichenko, IA Gak // Scientific Journal of the Ministry of Health of Ukraine. - 2014. - № 1 (5). - P. 93-99.

4. Litvinova EV Optimizing effect of laser therapy on the wound process and reactivity of the body of women with preeclampsia with preeclampsia / E.V. Litvinova / Archive of Clinical and Experimental Medicine. - 2003. - V. 12, № 1. - P. 57-61.

5. Яхъева М.Р. Features of lactation at women in labor of the Chechen Republic and ways of its optimization: the dissertation ... The candidate of medical sciences: 14.01.01 "Obstetrics and gynecology" / Yahyeva MR - Moscow, 2010. - 165 p.

6. Esartia MA Prediction, prevention and treatment of early disorders of lactation: dissertation abstract. ... Candidate of Medical Sciences: 14.00.01 "Obstetrics and Gynecology" / MA Esartia.- Samara, 2007.- 28 p .:

7. Yakovleva LV Estimation of physical development of children of the first year of life depending on a kind of feeding / L.V. Яковлева, А.А. Latypova, VR Башаров, Л.Р. Nurgalieva / Modern problems of science and education. - 2014. - № 3. Electronic resource: <http://www.science-education.ru/ru/article/view?id=13522>.

8. Омарова М.Ш. Lactation function at women at a combination of a hypothyroidism and an iron deficiency anemia: the dissertation ... The candidate of medical sciences: 14.00.01 "Obstetrics and gynecology" / Omarova M.Sh. - Rostov-on-Don, 2008. - 180 p.

9. Trusova O.Yu. Causes and risk factors of transfer of children to artificial feeding: the program of support of natural feeding: the dissertation ... The candidate of medical sciences: 14.00.09 "Pediatrics" / Trusova O.Yu. - 2007. - 261 p

10. Sergienko SN The pathogenetic role of the immune and microcirculatory disorders in chronic diseases of the hepatobiliary system in women of child-bearing age living in the industrial region of the Donets Basin / S.N. Sergienko // Lik Sprava. - 1997. - 6. - P. 45-48.

11. Markov AG The content of prostaglandin P2-alpha and prolactin in blood plasma and milk in women during lactogenesis / AG Markov, EN Pariyskaya, MA Kucherenko // Obstetrics and gynecology. - 2006. - № 2. - P.33-35.

12. Odent M.R. Synthetic oxytocin and breastfeeding: reasons for testing and hypothesis / M.R. Odent / Med Hypotheses. - 2013. - Vol. 81 (5). - P. 889-891.

13. Gerasimovich GI Function of lactation and breastfeeding / GI Gerasimovich // Health. - 2003. - № 11. - P. 26–33.

14. Nelyubova AB Hypogalactia. Diagnosis, prevention and treatment: dissertation abstract. ... cand. honey. Sciences: 14.01.01 "Obstetrics and gynecology" / AB Nelyubova. - Omsk, 2010. - 22 p.

15. Garunov EG Influence of an arterial hypotension on lactation function: the dissertation ... The candidate of medical sciences: 14.00.01 "Obstetrics and gynecology" / Garunov EG - Volgograd, 2006.

16. Bakun OV Hypogalactia as a manifestation of complications of the postpartum period / OV Bakun, VG | Kupchanko, A.M. Berbets, OA Andri-ets // Electronic resource: <http://dspace.bsmu.edu.ua:8080/xmlui/handle/123456789/1649>.

17. Countouris M.E. Effects of lactation on postpartum blood pressure among women with gestational hypertension and preeclampsia.E. Countouris, E.B. Schwarz, B.C. Rossiter [et al.] // Am. J. Obstet. Gynecol. - 2016. - pii: S0002-9378 (16) 00388-4.

18. Gutikova LV Hormonal regulation of lactation in women who have undergone preeclampsia / LV Gutikova // Journal of the Grodno State Medical University: a quarterly scientific-practical journal. - 2010. - №1. - P. 68-69

19. Marik P.E. Hypertensive disorders of pregnancy / P.E. Marik // Postgrad Med. - 2009. - Vol. 121 (2). - P. 69-76.

20. Olshevsky VS Neuroendocrine regulation of lactation function / VS Olshevsky. Olshevsky / Questions of experimental and clinical medicine: collection. Science. articles - 2012. - Vip. 16, vol. 2. - pp .176-183.

21. Wlodek M.E. Impaired mammary function and parathyroid hor-mone-related protein during lactation in growth-restricted sponta-neously hypertensive rats / M.E. Wlodek, K.T. Westcott, A. Serruto [et al.] // J Endocrinol. - 2003. - Vol. 178 (2). - P. 233-245.

22. Mangesi L. Treatments for breast engorgement during lactation / L. Mangesi, I. Zakarija-Grkovic // Cochrane Database Syst Rev. - 2016. - Vol. 28; (6). - CD006946.

23. Amirkhanova MI Disruption of lactation function in women who have given birth with preeclampsia: dissertation ... Candidate of Medical Sciences: 14.00.01 «Obstetrics and and gynecology" / M.I. Amirkhanova - Rostov-on-Don, 2002 .-- 147 p.

24. Abdullaeva M.Z. Features of gestation and lactation in young infants: abstract dis. ... candidate of medical sciences: 14.00.01 "Obstetrics and gynecology" / M.Z. Abdullaev. - Rostov-on-Don, 2007 .-- 2
