# Supplementary material for: Breast hypoplasia markers among women who report insufficient milk production: A retrospective online survey
Source: PLoS One. 2024 Feb 29;19(2):e0299642. doi: 10.1371/journal.pone.0299642 (PMC10903845; doi:10.1371/journal.pone.0299642)
Supplement: Attachment — Submitted filename: Response to Reviewers_20240201.docx [file pone.0299642.s016.docx]

**February 1^st^ 2024**

**Re: Revisions to Manuscript ID PONE-D-23-19510**

Dear Gilbert Sterling Octavius,

Many thanks to you and the reviewers for their assessment of our manuscript entitled "**Breast hypoplasia markers among women who report insufficient milk production: A retrospective online survey**”. We have addressed each of the required revisions and uploaded two versions of the revised manuscript – one with and one without tracked changes.

Kind regards,

Renee Kam and co-authors

**Editor comments:**

**Comment: 1. Please review your reference list to ensure that it is complete and correct. If you have cited papers that have been retracted, please include the rationale for doing so in the manuscript text, or remove these references and replace them with relevant current references. Any changes to the reference list should be mentioned in the rebuttal letter that accompanies your revised manuscript. If you need to cite a retracted article, indicate the article’s retracted status in the References list and also include a citation and full reference for the retraction notice.**

Response: No changes to reference list.

**Reviewer 1 comments:**

**Comment: Great job on the edits and additions, especially as a result of scrutinizing the youth weight data- you found some significant correlations that add valuable insight into the pathogenesis of hypoplasia.**

Response: Thank you!

**Comment: Page 5 line 72-77: The anatomical examples you are citing have another obvious potential mechanism of interference- latch/milk removal, that is not acknowledged--while the less likely possibility that these anatomical variations reflect glandular tissue is the implication left, especially as the next sentence mentions physiological changes that may reflect internal functioning. Is this your intent?**

Response: Thank you for raising this point. We have added an additional sentence as follows: “It is unclear if the poor breastfeeding outcomes were directly related to the anatomical issues or due to difficulties with infant latching to the breast and effectively removing milk.” (Page 5, lines 75-78)

**Comment: Page 6 line 110: I believe you mean to say the proportion of women with low supply who also have hypoplasia markers, rather than the proportion of women in general... perhaps amend this to "the proportion of this population who also have one or more various anatomical..." or similar**

Response: Thank you. We have edited this sentence to make it clearer as follows:

**Previously submitted manuscript:** “Therefore, using an online survey of women self-identifying as having low milk supply, we aimed to estimate the proportion of women with various anatomical breast characteristics related to breast hypoplasia, assess the feasibility of maternal self-report of these characteristics and to explore breast hypoplasia risk factors.”

**Revised manuscript: “**“Therefore, using an online survey of women self-identifying as having low milk supply, we aimed to estimate the proportion of this sample of women with various anatomical breast characteristics related to breast hypoplasia, assess the feasibility of maternal self-report of these characteristics and to explore breast hypoplasia risk factors.” (P 6, line 111)

**Comment: Page 22- bottom-- Pre-pregnancy BMI section appears twice?**

Response: Thank you, the duplicated pre-pregnancy BMI section in the previously submitted ‘Revised manuscript with Track Changes’ has been removed.

**Comment: Page 31: ‘The high proportion of women self-reporting low supply in this sample with various proposed breast hypoplasia markers cannot be determinative until compared to a reference population of women with normal milk production/general population.’ I am not sure you need “general population” here, or perhaps this needs some tweaking.**

Response: Thank you. We agree and have removed ‘/general population’ in the revised manuscript. (P 28, line 541)

**Comment: P 23 version 1 discussion: for future research, might I suggest adding the variable of percentage of milk produced to relate to the anatomical variables? Kuznetsov used the following for degrees of hypogalactia: I - milk deficit is less than 25% of daily volume; II - milk deficit is 26-50%; III - milk deficit is 51-75%; IV - milk deficit is greater than 75% [3]. Kuznetsov, V. (2017). Clinical and pathogenetic aspects of hypogalactia in post parturient women. Актуальні проблеми сучасної медицини: Вісник української медичної стоматологічної академії, 17(1 (57)), 305-307.**

**Thank you for this suggestion. We would like to add the following new sentence at the end of the Discussion “It would also be valuable to assess the relationship between deficit in maternal milk production (e.g. 75% deficit of daily volume [ref]) and breast hypoplasia markers.” However, we unfortunately have not been able to source the journal article and hence cannot cite it. We are therefore wondering if you could help us source it?

*Article and translation uploaded to you.**

Response: Thank you. We have included the additional sentence and reference at the end of the Discussion section. (P 29, lines 556-558)

**Reviewer 2 comments:**

**Comment: I am satisfied with attention to the comments of the reviewers, as responded to by changes or explanations.**

Response: Thank you
